# Standard-based comprehensive detection of adverse drug reaction signals from nursing statements and laboratory results in electronic health records

**DOI:** 10.1093/jamia/ocw168

**Published:** 2017-01-13

**Authors:** Suehyun Lee, Jiyeob Choi, Hun-Sung Kim, Grace Juyun Kim, Kye Hwa Lee, Chan Hee Park, Jongsoo Han, Dukyong Yoon, Man Young Park, Rae Woong Park, Hye-Ryun Kang, Ju Han Kim

**Affiliations:** 1 Division of Biomedical Informatics, Seoul National University College of Medicine, Seoul, Korea; 2 Department of Biomedical Sciences, Seoul National University Graduate School, Seoul, Korea; 3 Department of Medical Informatics and Internal Medicine, St. Mary Hospital, Catholic University, Seoul, Korea; 4 Cipherome Inc., Seoul, Korea; 5 Department of Biomedical Informatics, Ajou University School of Medicine, Suwon, Korea; 6 Mibyeong Research Center, Korea Institute of Oriental Medicine, Daejeon, South Korea; 7 Department of Internal Medicine, Seoul National University Hospital, Seoul, Korea

**Keywords:** pharmacovigilance, postmarketing surveillance, adverse drug reactions, algorithms, standard nursing statements, laboratory abnormalities

## Abstract

**Objective.** We propose 2 Medical Dictionary for Regulatory Activities–enabled pharmacovigilance algorithms, MetaLAB and MetaNurse, powered by a per-year meta-analysis technique and improved subject sampling strategy.

**Matrials and methods.** This study developed 2 novel algorithms, MetaLAB for laboratory abnormalities and MetaNurse for standard nursing statements, as significantly improved versions of our previous electronic health record (EHR)–based pharmacovigilance method, called CLEAR. Adverse drug reaction (ADR) signals from 117 laboratory abnormalities and 1357 standard nursing statements for all precautionary drugs (*n*  = 101) were comprehensively detected and validated against SIDER (Side Effect Resource) by MetaLAB and MetaNurse against 11 817 and 76 457 drug-ADR pairs, respectively.

**Results.** We demonstrate that MetaLAB (area under the curve, AUC = 0.61 ± 0.18) outperformed CLEAR (AUC = 0.55 ± 0.06) when we applied the same 470 drug-event pairs as the gold standard, as in our previous research. Receiver operating characteristic curves for 101 precautionary terms in the Medical Dictionary for Regulatory Activities Preferred Terms were obtained for MetaLAB and MetaNurse (0.69 ± 0.11; 0.62 ± 0.07), which complemented each other in terms of ADR signal coverage. Novel ADR signals discovered by MetaLAB and MetaNurse were successfully validated against spontaneous reports in the US Food and Drug Administration Adverse Event Reporting System database.

**Discussion.** The present study demonstrates the symbiosis of laboratory test results and nursing statements for ADR signal detection in terms of their system organ class coverage and performance profiles.

**Conclusion.** Systematic discovery and evaluation of the wide spectrum of ADR signals using standard-based observational electronic health record data across many institutions will affect drug development and use, as well as postmarketing surveillance and regulation.

## INTRODUCTION

A drug with demonstrated clinical efficacy in many patients can still be ineffective in other patients, or even cause serious side effects, including death.^1^^,^^2^ The incidence of severe adverse drug reactions (ADRs) has been estimated at 6.2–6.7% in hospitalized patients, and >2 million ADRs are reported annually in the United States, including 100 000 deaths.^1^^,^^3^ It is imperative to be vigilant for ADRs when administering all marketed drugs.^4^

The relevance of postmarketing pharmacovigilance has been growing steadily over the last 4 decades.^5^^,^^6^ The plethora of prescription, laboratory, and clinical information in electronic health record (EHR) systems has vast potential to drive diverse pharmacovigilance studies.^7^^,^^8^ Extracting ADR signals from laboratory results for specific medications has been the primary strategy of EHR-based pharmacovigilance studies, but they have evaluated only small numbers of drugs, ADRs, and their combinations. A comprehensive evaluation of all medications versus all laboratory results and all clinical narratives is still challenging, owing to a lack of efficient analytic algorithms, systematic evaluation strategies, and reliable reference standards for ADR signals.

Nurses reportedly play a more important role in discovering and spontaneously reporting ADRs than doctors and pharmacists.^9^^,^^10^ This may be due to nurses’ regular clinical observation/recording and more standardized statements compared to the diagnosis codes and test results recorded by doctors.^9–12^ Nursing records contain ADR signals in the form of clinical symptoms and signs, such as dizziness, dry mouth, and weight gain, that are not detectable by laboratory tests. Therefore, combining laboratory results with nursing statements could synergistically extend the usefulness of EHR-based pharmacovigilance. For all nursing documents, the Seoul National University Hospital (SNUH) EHR has applied standard nursing statements (SNSs) encoded by the International Classification for Nursing Practice (ICNP) for 10 years at the institutional level.

MetaLAB for laboratory results and MetaNurse for SNSs are significantly improved versions of our previous EHR-based pharmacovigilance algorithm, named CLEAR,^13^ powered by an advanced subject-sampling strategy for managing all drugs, all laboratory results, and all SNSs. A meta-analysis technique was applied to correct for yearly variations in drug-prescription and ADR-signal frequency patterns. Furthermore, to enable unbiased and comprehensive validation of ADR signals that are comprehensively detected,^14^ we created a comprehensive reference standard for ADR (RS-ADR), integrating and mapping Side Effect Resource 2 (SIDER 2)^15^ information and EHR data with standard biomedical vocabularies from the ICNP, International Classification of Diseases (ICD), Logical Observation Identifiers Names and Codes (LOINC), World Health Organization Adverse Reactions Terminology (WHOART), and Medical Dictionary for Regulatory Activities (MedDRA).

## METHODS

The ADR signals considered in this study were laboratory test results and SNSs in EHR data. This study was reviewed and approved by the SNUH Institutional Review Board, No. 1211-055-442.

### Data sources

**Figure 1.**
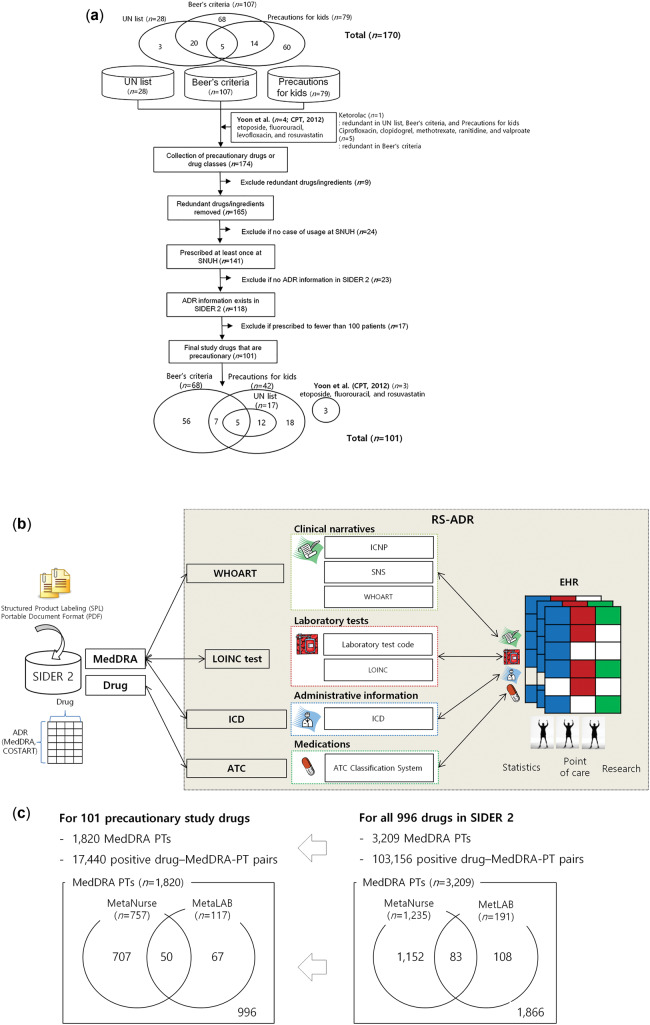
Data source and reference set for ADR signal detection and validation. (**A**) Inclusion and exclusion steps that yielded 101 precautionary study drugs. (**B**) The RS-ADR was created by standard vocabulary-based mapping of drug-ADR associations between SIDER 2 and EHRs. (**C**) Composition of ADRs annotated with MedDRA PTs detectable by MetaNurse and MetaLAB for all 996 SIDER 2 drugs (right panel) and 101 precautionary study drugs (left panel). UN, United Nations.

We analyzed all EHR data for inpatients obtained from January 1, 2005, to December 31, 2011, at SNUH, which is a tertiary teaching hospital with 1800 inpatient beds. Each SNUH EHR contains information on admissions, discharges, drug prescriptions, laboratory results, and nursing documents filled with SNSs encoded using ICNP terms at the enterprise level. We created a study database containing 82 935 010 prescriptions, 167 186 558 laboratory results, and 234 158 907 SNSs that covered 270 789 patients. ADR signals were detected by the 223 WHOART terms mapped to 1357 SNS terms and 117 laboratory abnormalities in MedDRA preferred terms (PTs). We extracted the records of all patients from the SNUH EHR database who had been prescribed at least one of the study drugs at least once during the study period (*n = *220 954) (Figure 1A), along with the laboratory results (*n = *91 171 636) and SNSs (*n = *74 488 476) from their admission and discharge notes.

Unlike previous studies that detected ADR by selecting only small numbers of specific drugs,^25^^,^^26^ we included all precautionary drugs in an unbiased manner. We collected all precautionary prescription drugs (*n* = 170) according to the Korean Food and Drug Administration’s recommendations, including the Beers criteria (*n* = 107),^16^^,^^17^ precautions for kids (*n* = 79),^18–20^ and the United Nations’ marketing prohibition drug list (*n* = 28)^21^^,^^22^ (Figure 1A). The 10 reference drugs reported by the CLEAR algorithm^13^ were also included so that a fair comparison could be made between the old and new algorithms. We excluded drugs that had redundant ingredients (*n* = 9), were not used at SNUH (*n* = 24), had no side effect information in SIDER 2 (http://sideeffects.embl.de/, released on March 16, 2012) (*n* = 23), and were prescribed to fewer than 100 patients at SNUH (*n* = 17), which finally yielded 101 study drugs for further investigation. The 101 drugs included in this study are classified according to the Anatomical Therapeutic Chemical (ATC) classification system and mapped to the product name used at SNUH (see Supplementary Table S2).

### Algorithms for ADR signal detection

**Figure 2.**
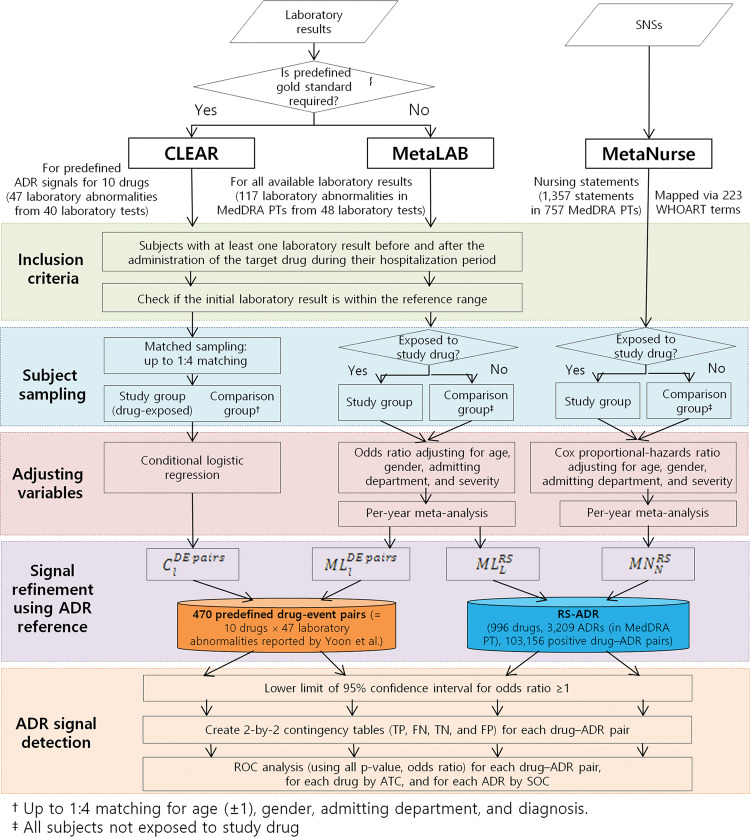
The analysis steps for the 3 ADR detection algorithms, CLEAR, MetaLAB, and MetaNurse. TP, true positive; FN, false negative; TN, true negative; FP, false positive.

Figure 2 illustrates the 5 steps of inclusion criteria, subject sampling, variable adjustment, signal refinement, and ADR signal detection used in the CLEAR,^13^ MetaNurse, and MetaLAB algorithms. The matched-sampling strategy of our previous CLEAR algorithm,^13^ involving up-to-1:4 matching for age, gender, admission department, and diagnosis, is prohibitively costly for large-scale applications that involve many drug-ADR pairs due to its computationally intensive and data-demanding nature. MetaLAB and MetaNurse utilized an improved strategy in which comparison groups were created by recruiting all subjects who were not exposed to the study drug, followed by variable adjustments for age, gender, admission department, and disease severity for computing the odds ratio for laboratory results (quantitative measurements) and Cox proportional-hazards ratio for nursing statements (frequency of ADR symptoms).

We found that the frequencies of drug prescriptions, laboratory tests, and nursing statements showed slow temporal fluctuations. This was overcome by controlling for yearly variations using the odds and Cox proportional-hazards ratios for each ADR per year from 2005 to 2011 at SNUH in a meta-analysis, which yielded integrated single odds and Cox proportional-hazards ratios, respectively, of that ADR over the study period. Meta-analysis was done using the meta metagen function of the R statistical package (version 4.3-0), using estimate of treatment effect and standard error of treatment estimate for each per year. MetaLAB was applied to the same 47 laboratory abnormalities from 40 laboratory tests used to evaluate CLEAR^13^ in a comparison study and also to all 117 laboratory abnormalities from 48 laboratory tests for the present study, to ensure that the evaluation was comprehensive (Figure 2 and Supplementary Table S3).

### 

#### CLEAR

CLEAR is a replication of our previous implementation for ADR signal detection^13^^,^^23^ applied to the laboratory results for the 47 laboratory abnormalities and validated against 470 predefined drug-event pairs, and is denoted as

$$C_{l}^{\text{DE pairs}},$$

where C stands for CLEAR algorithm, DE pairs corresponds to 470 drug-event pairs [=10 (drugs) × 47 (laboratory abnormalities)], and l is a label indicating the laboratory results.

#### MetaLAB

MetaLAB is an improved version of CLEAR. It includes a meta-analysis technique normalized on a yearly basis and an improved patient-sampling and comparison group–creation strategy. While CLEAR randomly matches each drug-exposed patient to up to 4 nonexposed patients by age (discrepancy of <1 year), gender, admitting department, and diagnosis, MetaLAB recruits all patients with no exposure to the drug as the comparison group without using matched sampling. The odds ratios are subsequently computed by adjusting for age, gender, admitting department, and disease severity. The disease severity of inpatients is determined twice daily by a nurse at SNUH. MetaLAB considers an abnormal laboratory result, defined as falling outside (higher or lower than) a certain reference range, as an ADR signal and computes the odds ratios of the ADR signals between the study and comparison groups. In addition, per-year odds ratios for each ADR from 2005 to 2011 at SNUH were input into a meta-analysis to obtain an integrated single odds ratio for the ADR over the study period. For CLEAR and MetaLAB, the observation periods for the study and comparison groups start at the first medication dose and second laboratory test dates, respectively, and continue until the discharge dates.

For the purpose of comparison with CLEAR, MetaLAB was applied to the same 10 drugs and validated against the same 470 drug-event pairs,^13^ denoted as

$${\text{ML}}_{l}^{\text{DE pairs}},$$

where ML stands for the MetaLAB algorithm, DE pairs corresponds to 470 drug-event pairs, and l is a label indicating the laboratory results tested using the 47 laboratory abnormalities. MetaLAB was also applied to all 101 precautionary drugs and validated against the more comprehensive RS-ADR, denoted as

$${\text{ML}}_{L}^{\text{RS}},$$

where ML stands for the MetaLAB algorithm, RS corresponds to the RS-ADR (referring to SIDER 2), and L is a label indicating the laboratory results tested by the 117 laboratory abnormalities.

#### MetaNurse

MetaNurse is an algorithm applied to SNSs (or frequency of ADR symptoms) instead of laboratory results (or quantitative measurements). MetaNurse determines an ADR signal for a drug when an SNS indicating the ADR is recorded more than twice after the first administration of the drug. Accordingly, we applied a Cox proportional-hazards ratio method by adjusting for age, gender, admitting department, and disease severity. The per-year Cox proportional-hazards ratios for each ADR from 2005 to 2011 at SNUH were input into a meta-analysis to obtain an integrated single Cox proportional-hazards ratio for the ADR over the study period. MetaNurse analysis is denoted as

$${\text{MN}}_{N}^{\text{RS}},$$

where MN stands for the MetaNurse algorithm, RS corresponds to the RS-ADR, and N is a label indicating the 1357 SNS terms mapped to 223 WHOART terms. The incidence of an ADR for a drug was defined as the number of patients having the ADR divided by the sum of the durations since the first medication and admission dates for the study and comparison groups, respectively, to the discharge date or date when the third WHOART terms annotated with SNS terms was recorded, whichever occurred earlier.

### Reference standard for adverse drug reactions

Given that there is no gold standard available for comprehensively and systematically validating pharmacovigilance studies, we created the RS-ADR by referring to the drug-ADR associations provided by the SIDER 2 database, which uses the MedDRA dictionary to extract ADR information from public documents and package inserts (Figure 1B). SIDER 2 provides information on 3209 recorded ADRs associated with 996 marketed drugs in MedDRA PTs. Only 103 156 drug–MedDRA-PT pairs (or 3.23% of all pairwise associations) are reported as positive drug-ADR associations in SIDER 2^15,^^24^ (Figure 1C, right panel). Most resources for drug-ADR associations including SIDER 2 do not provide negative associations, only positive ones. The presence of “no report” or “not found” entries in these resources does not necessarily mean a true-negative association. Moreover, correct evaluations are almost always hampered when there is no gold standard for true negatives. This is one reason why previous studies focused on small numbers of drugs and/or ADRs. In the absence of a true gold standard for drug-ADR associations, we used the SIDER 2 set as the positive reference set for the purpose of current validation.

Figure 1B illustrates the steps used to create the RS-ADR by mapping (1) both clinical narratives and ADRs encoded by SNS terms and MedDRA PTs, respectively, to WHOART, (2) SNUH codes for laboratory tests to LOINC, and then LOINC terms and SNUH codes to MedDRA PTs, and (3) administrative classifications for ICD codes to MedDRA PTs. We manually mapped SNS terms (encoded by the ICNP) and MedDRA PTs to WHOART. Presumed drug-ADR associations that do not yet exist in SIDER 2 but are implicitly suggested by this database were explicitly connected as established ADRs. In other words, when a group of MedDRA PTs were mapped to the same WHOART term but only a portion of the PT group was assigned to a drug-ADR association by SIDER 2, all MedDRA PTs in that group were explicitly assigned to the drug as established ADRs. For example, among 5 MedDRA PTs mapped to the same WHOART term (eg, *Urticaria*, *Urticaria chronic*, *Urticaria physical*, *Mechanical urticaria*, and *Urticaria vesiculosa*), only *Urticaria* was reported as an ADR of ranitidine in SIDER 2. We linked the remaining 4 PTs to ranitidine as its ADRs in the RS-ADR (see Supplementary Table S1). Filling the gaps between implicit drug-ADR associations using standard ADR terminologies such as WHOART is a critical step when building a reference standard for ADRs; for example, a correct prediction of the ranitidine–*Urticaria physical* association by a pharmacovigilance algorithm might be evaluated to be incorrect (ie, a false positive). These mapping and manual curation processes for building the RS-ADR in the present study took >2 years and were validated by 3 clinical experts. A detailed description of the RS-ADR will be reported separately.

Of the 3209 MedDRA PTs for 996 drugs in SIDER 2, 1235 MedDRA PTs were mapped by the RS-ADR to 1439 SNS terms by 239 WHOART PTs for MetaNurse and 191 laboratory abnormalities from 62 laboratory tests for MetaLAB. Of the 1820 MedDRA PTs in 17 740 positive drug–MedDRA-PT pairs for the 101 precautionary drugs in the RS-ADR, 757 MedDRA PTs were mapped to 1357 SNS terms by 223 WHOART PTs for MetaNurse and 117 laboratory abnormalities from 48 laboratory tests for MetaLAB (via LOINC) (Figures 1C and 2).

### Evaluation

Logistic regression and Cox proportional-hazards models were applied to calculate the odds ratios and 95% confidence intervals between drug prescriptions and ADR signals indicated by abnormal laboratory results and SNSs, respectively (Figure 2). To identify unknown but significant ADRs, we used the 95% confidence interval with a lower limit ≥1.0 for a drug–adverse event pair as a positive ADR signal, as in our previous study.^13^

For calculating AUC (area under the receiver operating characteristic curve [ROC]), we used a meta-regression method widely used in meta-analysis for obtaining ROC curves.^25^^,^^26^ The epicalc lroc function of the R statistical package (version 2.15.1.0) creates ROC curves directly from a logistic regression model that are applied to multiple comparisons. We applied adjusted P-values (by the Benjamini-Hochberg method) and binary outcomes (whether the ADRs of the drug were known or not, based on the outcomes listed in SIDER 2) together with odds ratios for MetaLAB or hazard ratios for MetaNurse as input numeric vectors to the epicalc lroc function for evaluating the performance of the 3 algorithms across different MedDRA system organ classes (SOCs) and ATC drug classes (Table 1).

**Table 1.** Clinical characteristics of the included subjects and precautionary drug exposure

Algorithm	CLEAR $C_{l}^{\text{DE pairs}}$	MetaLAB ${\text{ML}}_{l}^{\text{DE pairs}}$	MetaLAB ${\text{ML}}_{L}^{\text{RS}}$	MetaNurse ${\text{MN}}_{N}^{\text{RS}}$
Gold standard for ADRs	DE pairs^a^		RS-ADR	
Type of ADR signals	Predefined laboratory abnormalities		MedDRA PTs	
No. of target drugs	10		101	
No. of ADR signals	47 (from 40 tests)		117 (from 48 tests)	757 (from 1357 SNSs)
No. of drug-ADR pairs	470		11 817	76 457
No. of positive pairs^b^	221		2210	34 857
No. of negative pairs^c^	249		9607	41 600
AUC, not integrated^d,^^e^	0.55 ± 0.06	0.61 ± 0.18	0.69 ± 0.11	0.62 ± 0.07
AUC, SOC-integrated^f^	–	–	0.84 ± 0.13	0.84 ± 0.09
No. of patients	68 769	88 038	215 088	220 954
No. of exposure cases^g^	90 804	127 171	1 028 724	1 187 037
Age (years)^h^	51.3 ± 18.9	50.7 ± 19.9	46.5 ± 23.0	46.22 ± 23.1
Female, *n* (%)^i^	38 290 (55.67)	47 470 (53.91)	108 410 (50.40)	110 864 (50.17)
Disease severity, *n* (%)^j^				
1	22 755 (33.08)	29 706 (33.74)	106 997 (49.74)	111 410 (50.42)
2	29 225 (42.49)	37 611 (42.72)	82 306 (38.26)	83 422 (37.75)
3	10 485 (15.24)	12 619 (14.33)	19 238 (8.94)	19 824 (8.97)
4	3306 (4.80)	4167 (4.73)	4130 (1.92)	4025 (1.82)
5	2882 (4.19)	3747 (4.25)	2304 (0.01)	2174 (0.09)
6	116 (0.16)	188 (0.21)	113 (0.0005)	99 (0.0004)

^a^Predefined DE pairs for 10 drugs and 47 laboratory abnormalities reported by Yoon et al.^13^

^b^Positive pairs were established by expert review, and the remaining pairs were considered to be ^c^Negative DE pairs.

^d^AUCs were computed by considering all the drugs and ADRs as a single dataset.

^e^CLEAR significantly outperformed MetaCLEAR (DeLong’s test for 2 ROC curves, P = .0137).

^f^AUCs were computed for each MedDRA SOC by stratifying ADRs and then integrating.

^g^Numbers of patient exposures to target drugs were summed by separately counting the exposures of each patient to different target drugs.

^h^Age differed significantly between CLEAR and MetaLAB (*P* = .01) and between MetaLAB and MetaNurse (*P* = 4.68 × 10^–7^) in Student *t* test.

^i^Gender did not differ significantly between the comparison groups (*P* = .89 and .93).

^j^Disease severity did not differ significantly between CLEAR and MetaLAB (*P* = .029), but it did differ significantly between MetaLAB and MetaNurse (*P* = 3.09 × 10^–6^) in Fisher’s exact test.

Supplementary Table S5(a) shows the variables used for calculating an AUC value for the drug atropine as an example. We used the epicalc lroc function in R statistical package to obtain ROC curves. The binary outcome was whether the ADRs of the drug were known or not, based on the outcomes in SIDER2. After obtaining AUC values for each of the 101 drugs against 117 laboratory abnormalities and 1357 SNSs in 757 MedDRA PTs for MetaNurse and MetaLAB, respectively, we obtained the overall AUC values by calculating the averages and standard deviations of the AUCs of the 101 drugs (Table 1).

We also computed AUC values for each of the 101 drugs per each SOC subgroup (Supplementary Table S5(b)). Because SIDER 2 does not provide negative associations, only positive ones, the nonreported drug-ADR associations are regarded as the reference-negative associations for evaluating massively detected ADR signals. The much bigger negative (than positive) association space of SIDER 2 creates challenging bias, severely overestimating the performance of algorithms that prefer negative calls, and vice versa. By restricting the search space to each SOC, the spurious negative (or nonreported by SIDER 2) drug-ADR associations in the reference set were significantly reduced without affecting SIDER-reported positive associations. Finally, we obtained the SOC-integrated AUC value for each of the 101 drugs by calculating the weighted average and standard deviation of the per-SOC AUC values (Table 1). These can also be used to compute the overall AUC values for each SOC by calculating the average and standard deviation of the per-SOC AUC values of the 101 drugs (Table 2).

**Table 2.** Performance of MetaLAB and MetaNurse for different MedDRA SOCs as measured by AUCs

SOC	MetaLAB				MetaNurse			
	AUC	Drugs with positive drug-ADR pairs (%)	No. of drug-ADR pairs		AUC	Drugs with positive drug-ADR pairs (%)	No. of drug-ADR pairs	
			No. of positive pairs	No. of negative pairs			No. of positive pairs	No. of negative pairs
Blood and lymphatic system disorders	0.79 ± 0.11	83.17	964	1,763	0.83 ± 0.16	84.16	505	606
Endocrine disorders	0.87 ± 0.15	49.50	90	516	0.87 ± 0.17	80.20	532	882
Hepatobiliary disorders^*^	0.95 ± 0.11	42.57	77	428	1.00 ± 0.00	60.40	752	662
Investigations	0.69 ± 0.12	88.12	727	4222	0.72 ± 0.12	97.03	2168	3791
Metabolism and nutrition disorders	0.71 ± 0.13	53.47	236	2188	0.78 ± 0.15	93.07	448	1,370
Renal and urinary disorders^*^	1.00 ± 0.00	35.64	115	390	0.82 ± 0.15	96.04	1624	2214
Cardiac disorders					0.74 ± 0.14	96.04	2415	2534
Ear and labyrinth disorders					0.97 ± 0.10	89.11	270	437
Eye disorders					0.80 ± 0.13	99.01	2739	2412
Gastrointestinal disorders					0.73 ± 0.11	98.02	4164	4522
General disorders and administration site conditions					0.68 ± 0.11	98.02	1668	1665
Immune system disorders					0.77 ± 0.15	97.03	881	634
Infections and infestations					0.82 ± 0.15	98.02	3985	3186
Musculoskeletal and connective tissue disorders					0.83 ± 0.12	98.02	904	813
Nervous system disorders					0.67 ± 0.12	99.01	2507	3654
Psychiatric disorders					0.71 ± 0.16	99.01	3767	4242
Reproductive system and breast disorders					0.95 ± 0.11	93.07	371	639
Respiratory thoracic and mediastinal disorders					0.78 ± 0.11	94.06	1421	2821
Skin and subcutaneous tissue disorders					0.80 ± 0.12	99.01	1978	1961
Vascular disorders					0.75 ± 0.15	96.04	1507	1927

Among 26 MedDRA SOCs, 6 having fewer than 5 MedDRA PTs for MetaNurse were omitted: congenital, familial, and genetic disorders (n = 3); injury, poisoning, and procedural complications (n = 1); neoplasms benign, malignant, and unspecified (n = 3); pregnancy, puerperium, and perinatal conditions (n = 1); social circumstances (n = 1); and surgical and medical procedures (n = 0). *P < .05 by paired t test. AUC data are mean ± SD values.

## RESULTS

### Algorithm performance

Table 1 and Figure 1 show the clinical characteristics of the study population and precautionary drug inclusion. Patient records were eligible for CLEAR^13^ and MetaLAB if the patients had been prescribed at least one target drug at least once and had one or more laboratory result before and after being administered the target drug during the hospitalization period^13^ (Figure 2). Patient records were eligible for MetaNurse if the patients had been prescribed at least one target drug at least once and had one or more SNSs in their EHRs (Figure 2). Table 1 (left panel) shows that MetaLAB (${\text{ML}}_{\text{l}}^{\text{DE pairs}}$, AUC = 0.61 ± 0.18) outperformed CLEAR ($C_{l}^{\text{DE pairs}}$, AUC = 0.55 ± 0.06) when we applied the same 470 drug-event pairs as the gold standard (Figure 2, left panel) as in our previous research.^13^ Correction for multiple hypothesis testing was performed by the Benjamini-Hochberg method provided by the p.adjust function in R statistical package. MetaLAB significantly outperformed CLEAR (Table 1, P = .0137 by DeLong’s test for 2 ROC curves).

**Figure 3.**
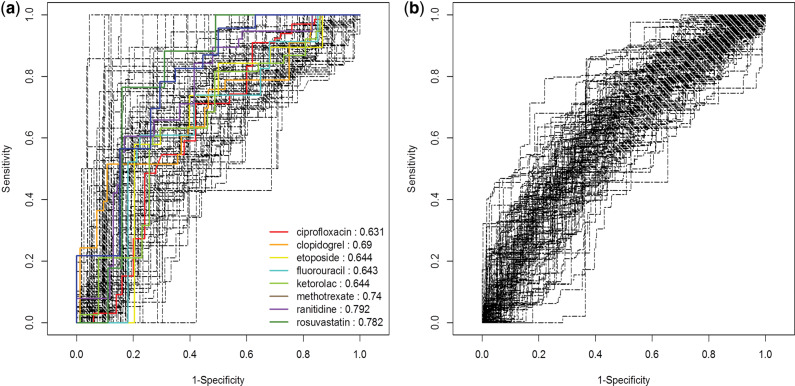
Evaluation of the ROC curves of pharmacovigilance algorithms created by (**A**) MetaLAB (${\text{ML}}_{L}^{\text{RS}}$, AUCs = 0.69 ± 0.11) and (**B**) MetaNurse (${\text{MN}}_{N}^{\text{RS}}$, AUCs = 0.62 ± 0.07) against the extensive RS-ADR gold standard comprising 117 and 757 MedDRA PTs, respectively, for 101 precautionary drugs. ROC curves for the 10 drugs reported by Yoon et al.^13^ for MetaLAB analysis against 11 817 drug-ADR pairs are presented as colored curves.

Figure 3 shows ROCs for 101 precautionary drugs against 117 laboratory abnormalities and 757 nursing statements in MedDRA PTs obtained by MetaLAB and MetaNurse (${\text{ML}}_{L}^{\text{RS}}$, 0.69 ± 0.11; ${\text{MN}}_{N}^{\text{RS}}$, 0.62 ± 0.07), respectively (Table 1). Despite the improved performance of MetaLAB compared to the previous implementation in CLEAR, the ROC values are modest (<0.70). SOC-integrated AUC computation greatly improved the performance of MetaLAB and MetaNurse (0.84 ± 0.13 and 0.84 ± 0.09, respectively) by separately obtaining an ROC value for each SOC for a drug and then averaging them to obtain the SOC-integrated ROC value for the drug. Otherwise, many SOCs with no signal can hamper the overall performance of an ADR signal detection algorithm, especially when evaluating vastly extended ADR space across all SOCs. Drugs tend to have ADRs enriched in specific SOCs only. For example, phenylephrine’s known side effects (*n* = 4) of laboratory abnormalities are reported only in blood and lymphatic system disorder (*n* = 2) and investigation (*n* = 2) SOCs (Supplementary Figure S3). Figure 3A shows the ROCs by MetaLAB for the same 10 drugs reported by Yoon et al.^13^ against the extensive 11 817 drug-ADR pairs in RS-ADR. Despite the vastly extended problem space, MetaLAB showed remarkably improved performance (0.69 ± 0.11, Table 1). Due to its dependence on a predefined gold standard,^13^ CLEAR cannot be tested against all 11 817 drug-ADR pairs but only against the predefined 470 pairs (Table 1). MetaLAB outperformed CLEAR when evaluated against the same 10 drugs and 47 laboratory abnormalities (${\text{ML}}_{l}^{\text{DE pairs}}$, 0.61 ± 0.18, Table 1).

### System organ class coverage

Standard vocabulary-based mapping enables systematic evaluation of ADR signals across different SOCs and ATC drug classes (Supplementary Figure S1). Six SOCs were covered by both MetaLAB and MetaNurse, but 14 can only be covered by MetaNurse (Table 2). It should be noted that skin and subcutaneous tissue disorders (AUC = 0.80 ± 0.12), nervous system disorders (AUC = 0.67 ± 0.12), and psychiatric disorders (AUC = 0.71 ± 0.16) are ADRs that are observed very often in clinical settings and are detectible with high prediction accuracy by MetaNurse but not by MetaLAB. Supplementary Figure S2 shows prediction performance profiles of MetaLAB and MetaNurse.

Among 26 MedDRA SOCs, 6 having fewer than 5 MedDRA PTs for MetaNurse were omitted: congenital, familial, and genetic disorders (*n* = 3); injury, poisoning, and procedural complications (*n* = 1); neoplasms benign, malignant, and unspecified (*n* = 3); pregnancy, puerperium, and perinatal conditions (*n* = 1); social circumstances (*n* = 1); and surgical and medical procedures (*n* = 0). ^*^P < .05 by paired *t* test. AUC data are mean ± SD values.

As shown in Table 1, SOC subgroup–integrated AUC values are bigger for MetaLAB (0.84 ± 0.13) and MetaNurse (0.84 ± 0.09) than the overall AUC values (${\text{ML}}_{L}^{\text{RS}}$, 0.69 ± 0.11; ${\text{MN}}_{N}^{\text{RS}}$, 0.62 ± 0.07, Table 1). While MetaNurse shows lower overall performance than MetaLAB, much wider ADR domains of MedDRA SOCs are covered by MetaNurse than by MetaLAB (Table 2). The AUC values measured for individual SOCs (Table 2) tend to be bigger than those measured for overall SOCs (Table 1), simply because a drug-ADR space is defined by the numbers of drugs and ADRs that are counted. The increment by SOC subgroup integration was larger for MetaNurse (0.22), covering more SOCs, than for MetaLAB (0.15) (Tables 1 and 2 and Supplementary Figure S1).

### Novel ADR signals

**Figure 4.**
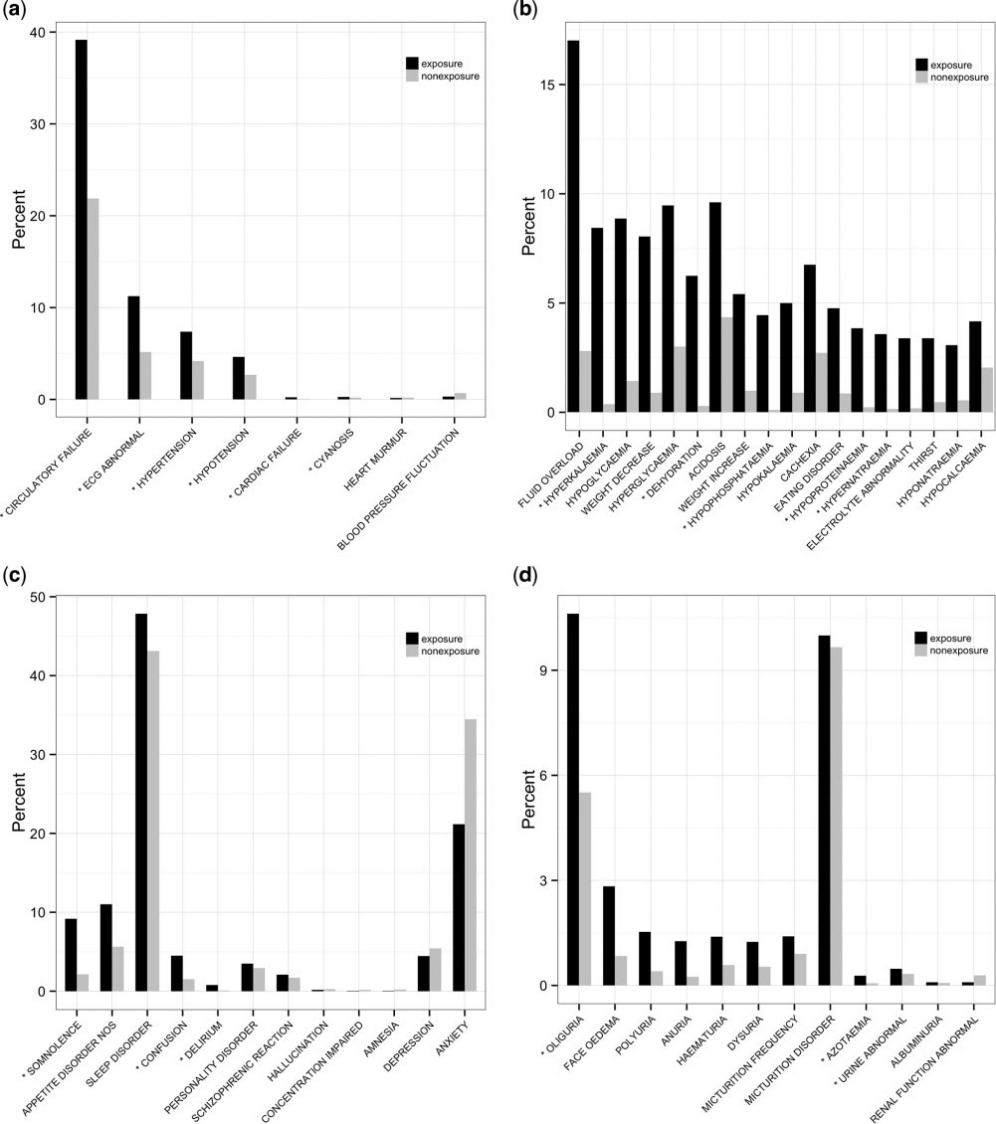
Distribution of ADR-signal frequency ratios between exposure and nonexposure groups. SOCs of (**A**) bisacodyl in cardiac disorders, (**B**) prazosin in metabolism and nutrition disorders, (**C**) phenylephrine in psychiatric disorders, and (**D**) sucralfate in renal and urinary disorders.

Positively predicted signals for unknown ADRs (or false positives) can be applied for novel ADR discovery. To evaluate false-positive ADR signals, spontaneous reports in the FDA Adverse Event Reporting System (FAERS)^27^ were annotated with MedDRA and WHOART PTs. Table 3 and Figure 4 exhibit evaluation examples for false-positive signals of 4 exemplar drugs: bisacodyl, prazosin, phenylephrine, and sucralfate.

**Table 3.** Adverse drug reactions reported in the FAERS database (submitted to FDA)

Drug (SOC)	No. ADRs No. (%) of reports in FAERS database	ADR signals stratified by number of reports						
		<3 reports	3–5 reports	6–10 reports	11–30 reports	31–50 reports	51–100 reports	>100 reports
Bisacodyl (Cardiac disorders)	39 ADRs in 354 of 14 645 reports (2.44%)	Atrial hypertrophy, cardioactive drug level above therapeutic, cardiorenal syndrome, cardiopulmonary failure, cardiac pacemaker replacement, cardiolipin antibody positive, cardiac pacemaker insertion, cardiac flutter, cardiac output decreased, cardiac failure chronic, cardiac myxoma, conduction disorder (*n* = 12)	Atrial tachycardia, ventricular hypertrophy, ventricular hypokinesia, cardiac asthma, cardiac failure acute, cardioactive drug level increased, cardiac valve disease, ventricular dysfunction, cardiac tamponade, dyspnea paroxysmal nocturnal (*n* = 10)	Ventricular fibrillation, cardiomyopathy, cardiogenic shock, ventricular extrasystole, dyspnea exertional, cardiac murmur, cardiovascular disorder (*n* = 7)	Cardiac arrest, cardiac failure, cardiomegaly, arrhythmia, atrial flutter, cardiac disorder, ventricular tachycardia (*n* = 7)	Atrial fibrillation, cardiorespiratory arrest (*n* = 2)	Cardiac failure congestive (*n* = 1)	
Prazosin (Metabolism and nutrition disorders)	6 ADRs in 110 reports (1.45%)	Hypernatremia, hypophosphatemia (*n* = 2)			Weight decreased, dehydration, decreased appetite (*n* = 4)	Hyperkalemia (*n* = 1)		
Phenylephrine (Psychiatric disorders)	14 ADRs in 398 reports (4.29%)	Confusion postoperative, mental disorder due to a general medical condition, consciousness fluctuating (*n* = 3)	Disorientation, mental impairment (*n* = 2)	Somnolence, mental disorder, delirium (*n* = 3)	Fear of death, confusional state, mental status changes, psychiatric symptom (*n* = 4)		Depression (*n* = 1)	Emotional distress (*n* = 1)
Sucralfate (Renal and urinary disorders)	65 ADRs in 705 reports (1.99%)	Urine output increased, nephritis, urinary tract infection pseudomonal, bladder spasm, bladder cancer, ureteric obstruction, renal cancer metastatic, renal artery arteriosclerosis, renal tubular acidosis, urethral stenosis, urine color abnormal, urine chloride decreased, urine bilirubin increased, urine calcium decreased, urinary bladder hemorrhage, urinary bladder rupture, urinary tract infection fungal, glomerulonephritis, glomerulonephritis proliferative, bladder pain, bladder neoplasm, bladder dilatation, bladder diverticulum, bladder obstruction, bladder irritation, urge incontinence, renal tubular disorder, renal stone removal, renal necrosis, renal pain, renal hematoma, renal cortical necrosis, renal atrophy, renal arteriosclerosis, renal ischemia (*n* = 35)	Proteinuria, bladder disorder, renal function test abnormal, renal mass, renal artery stenosis, renal cancer, urine analysis abnormal, urinary tract obstruction, bladder prolapse, renal hemorrhage, renal transplant (*n* = 11)	Chromaturia, azotemia, tubulointerstitial nephritis, oliguria, renal cell carcinoma, urinary hesitation, urinary tract disorder (*n* = 7)	Renal disorder, renal impairment, urinary retention, renal cyst, urinary incontinence, renal tubular necrosis, urine output decreased, renal injury (*n* = 8)	Renal failure chronic (*n* = 1)		Renal failure, renal failure acute, urinary tract infection (*n* = 3)

While bisacodyl’s ADRs are mainly classified into the *Gastrointestinal disorders* SOC,^28^ MetaNurse detected 25 MedDRA PTs that are unknown but significant in the *Cardiac disorders* SOC (lower limit of 95% CI ≥ 1.0) and can be nonredundantly mapped to 6 WHOART PTs, *Circulatory failure*, *ECG abnormality*, *Hypertension*, *Hypotension*, *Cardiac failure*, and *Cyanosis* (Figure 4A). Cardiac disorders are not currently known as bisacodyl ADRs, according to Micromedex^™^. However, we found that 354 (2.44%) of the 14 645 FAERS reports for bisacodyl^29^ were associated with cardiac abnormalities, including 55 congestive heart failure (CHF), 49 atrial fibrillation, and 31 cardiorespiratory arrest reports (Table 3).

Supplementary Table S4 shows that the baseline or average rate of cardiac abnormalities for all drugs in the FAERS database was 2.21%, which was lower than bisacodyl’s 2.44%. Moreover, docusate sodium and polyethylene glycol 3350, which are known to have side effects involving cardiac abnormalities, showed 2.48% and 1.55%, respectively, and lactulose and senna, which have no known side effects involving cardiac abnormalities, showed 1.93% and 1.95%, respectively. Based on these findings, we concluded that the risk of cardiac abnormalities from bisacodyl is higher than the baseline risk.

Supplementary Figure S4 shows that the statistical significance of the 6 WHOART PTs, circulatory failure, ECG abnormality, hypertension, hypotension, cardiac failure, and cyanosis (Figure 4A), were not affected even if we excluded the patients with CHF, for whom laxatives are sometimes prescribed to minimize straining. We performed a detailed analysis of the bisacodyl case by controlling the ICD-10 code for CHF, I-50*. A case with CHF was defined as a patient having a diagnosis code of I-50* before being prescribed bisacodyl. Because only 0.41% had CHF, the overall statistical significance was not affected. CHF was diagnosed in 912 ( = 268 + 644, 0.41%) but not in 220 042 ( = 45 143 + 174 692, 99.59%) patients. Diagnosis of CHF was made before bisacodyl was prescribed according to the analysis steps in our algorithms.

For prazosin, for which known ADRs are gastrointestinal and cardiovascular,^30^ MetaNurse detected 5 novel ADR signals in the metabolism and nutrition disorders SOC not known to Micromedex^™^: hyperkalemia, dehydration, hypophosphatemia, hypoproteinemia, and hypernatremia. Of the 7555 FAERS reports for prazosin,^31^ 110 (1.45%) were associated with metabolism and nutrition, including 33 on hyperkalemia, 29 on weight decreased, 26 on dehydration, and 19 on decreased appetite.

For phenylephrine,^32^ MetaNurse detected 3 novel ADR signals in the psychiatric disorders SOC that are not known to Micromedex^™^, somnolence, confusion, and delirium. Of the 9332 FAERS reports for phenylephrine, 398 (4.29%) were associated with psychiatric problems.^33^

Gastrointestinal ADRs are common with sucralfate,^34^ but MetaNurse detected 3 novel signals in the renal and urinary disorders SOC that are not known to Micromedex^™^, oliguria, urine abnormality, and azotemia. Of the 34 985 FAERS reports for sucralfate,^35^ 705 (1.99%) were associated with renal and urinary problems.

## DISCUSSION

MetaLAB and MetaNurse are improved versions of our previous CLEAR algorithm^13^ powered by an advanced subject-sampling strategy, a meta-analysis technique that adjusts for yearly variations in drug prescriptions and/or disease prevalence, and a comprehensive reference standard for detecting ADR signals. They assign the records of all patients who are not prescribed the study drug as the comparison group and adjust confounding factors (Figure 2). MetaLAB outperformed CLEAR (Table 1).

Most of the previous studies focused on a small number of preselected ADRs such as prolonged QT interval, myocardial infarction, cardiac valve fibrosis, and venous thrombosis.^36^^,^^37^ In our previous study^13^ where we developed a reference set for the CLEAR algorithm, experts had to manually create and curate a mapping table linking known ADRs and laboratory abnormalities (interobserver agreement κ = 0.95; *P* < .001). For the present study, we created a comprehensive ADR knowledge base, called the RS-ADR, referring automatically to SIDER 2, to be applied to all commercially available drugs and to all nursing statements and laboratory abnormalities. Moreover, we mapped SIDER 2 information and EHR data with controlled vocabularies. This mapping of EHR data to standard controlled vocabularies was reviewed by 3 clinical experts, with a high degree of interobserver agreement (κ = 0.84; *P* < .001). Further information about the RS-ADR will be presented in a separate report.

Only a few studies of ADR signals have applied standard biomedical vocabularies.^11^^,^^38^^,^^39^ Increasing the use of controlled vocabularies in EHR systems will enable users to easily search and compare clinical symptoms, signs, procedures, treatments, and test results that contain trigger signals associated with ADRs. We have integrated MedDRA, WHOART, and ATC drug classes with controlled vocabulary-annotated EHR data to systematically analyze ADR signals.

The increasing use of coded nursing statements in EHRs provides an additional opportunity to improve EHR-based pharmacovigilance. The present study demonstrates the symbiosis of laboratory test results and nursing statements for ADR signal detection in terms of their different SOC coverages and performance profiles. Nursing statements contain more standardized and consistent information on dimensions compared to laboratory results. Our use of SNSs makes it possible to detect ADR signals over a wide range of clinical symptoms, such as dermatitis, eyelid ptosis, and sleep disorders. For example, MetaLAB showed high performance for nicardipine (AUC = 0.84), clopidogrel (AUC = 0.69), and lorazepam (AUC = 0.61), whose major ADRs are hypokalemia, leukocytosis, anemia, and abnormal liver function test, which are easily detectible by laboratory tests. In contrast, MetaNurse showed high performance for tolterodine (AUC = 0.79) and mirtazapine (AUC = 0.72), whose major ADRs are diarrhea, anaphylactic shock, and edema, which are more likely to be detected by bedside nursing observations (data not shown).

Pharmacovigilance algorithms can be used to discover novel ADR signals. With improved performance and extended coverage for drugs and ADR signals, we discovered numerous significant ADR signals that had not been identified previously using SIDER 2 and Micromedex^™^ (Table 3). In particular, using clinical observations in nursing statements greatly extended the search space for ADR signals for many SOCs, such as skin and subcutaneous-tissue disorders, nervous system disorders, and psychiatric disorders (Table 2 and Supplementary Figure S1). Supplementary Figure S2 shows that MetaNurse identifies more novel ADR signals (or false positives) in many SOCs and ATC drug classes than MetaLAB does. Our comprehensive analysis results for all 101 precautionary drugs by MetaNurse and MetaLAB for 22 MedDRA and 7 SOCs are available for further validation by users at our website http://adr.snubi.org/.

The present study was subject to some limitations. First, dose-related ADRs were not considered; this would require a database with information on dose-related ADRs, which is not currently available. However, it may be possible to integrate dosing information with our algorithms for a limited number of ADRs whose dose-related effects are well established. Second, the causality between drug exposure and detected ADR signal was not verified. It is necessary to establish causality, since this evaluates the relationship between a drug treatment and the occurrence of an adverse event.^40^ Third, comparing the performance of MetaLAB and MetaNurse in ADR signal detection was not straightforward, because there is no established gold standard for validating ADR signal-detection algorithms. We chose to use SIDER 2 as the “silver” standard for performance evaluations. Fourth, some hospitals are not yet using standard nursing statements. However, the results obtained in the present study should encourage the use of coded nursing statements in practice. It is suggested that simple text mining and natural language processing of clinical narratives, including nursing statements, can greatly reduce the incidence of ADRs. Fifth, our subject sampling and matching strategy based on drug exposure vs nonexposure groups could be vulnerable to misinterpretation of drug indications as ADR signals. However, our strategy has the advantage of increasing the study population size, hence compensating for this drug-indication bias to a certain degree, as shown in Supplementary Figure S4.

## Author Contributions

SHL, JC, HSK, GJK, HRK, and JHK wrote the manuscript and contributed to revision of the paper. SHL, JC, KHL, DY, MYP, RWP, and JHK contributed to the conception and design of the study. SHL, JC, KHL, JH, and JHK performed the research. SHL, JC, and JHK analyzed the data.

SHL, JC, CHP, and JHK contributed new reagents/analytical tools.

## Funding

This work was supported by the grants of the Korean Health Technology R&D Project, Ministry of Health and Welfare (HI16C1128) and of the Ministry of Food and Drug Safety(16172MFDS170). JHK would like to gratefully acknowledge the Education and Research Encouragement Fund of Seoul National University Hospital.

## Competing interests

No competing interests are identified by the authors of this manuscript.

## SUPPLEMENTARY MATERIAL

Supplementary material is available at *Journal of the American Medical Informatics Association* online.

## Supplementary Material

Supplementary Data
