# Supplementary material for: Standard-based comprehensive detection of adverse drug reaction signals from nursing statements and laboratory results in electronic health records
Source: J Am Med Inform Assoc. 2017 Jan 13;24(4):697–708. doi: 10.1093/jamia/ocw168 (PMC7651894; doi:10.1093/jamia/ocw168)
Supplement: Supplementary Data [file ocw168_supp.zip › Supplementary_Table_S1_r.docx]

**Supplementary Table S1.** SNS terms encoded by the ICNP and mapped to WHOART and MedDRA PTs.

| MedDRA PT | WHOART | SNS terms at SNUH | ICNP |
| --- | --- | --- | --- |
| *Urticaria*  *Urticaria chronic*  *Urticaria physical*  *Mechanical urticaria*  *Urticaria vesiculosa* | *Urticaria* | Urticarial decreased | “detail,” “body site,” “abnormal skin lesion,” “decreased” |
|  |  | Urticarial not changed | “detail,” “body site,” “abnormal skin lesion,” “not changed” |
|  |  | Urticarial positive | “detail,” “body site,” “abnormal skin lesion,” “yes” |
|  |  | Urticarial increased | “detail,” “body site,” “abnormal skin lesion,” “increased” |
