# Supplementary material for: Standard-based comprehensive detection of adverse drug reaction signals from nursing statements and laboratory results in electronic health records
Source: J Am Med Inform Assoc. 2017 Jan 13;24(4):697–708. doi: 10.1093/jamia/ocw168 (PMC7651894; doi:10.1093/jamia/ocw168)
Supplement: Supplementary Data [file ocw168_supp.zip › Supplementary_Table_S2_a_r.docx]

| **Supplementary Table S2** 101 precautionary study drugs lists  **(a)** ATC classification of the 101 drugs applied to MetaLAB and MetaNurse. | | | | |
| --- | --- | --- | --- | --- |
|  |  |  |  |  |
| **ATC classification** | **No. of drugs** |  |  |  |
| Alimentary tract and metabolism (A) | 21 |  |  |  |
| Blood and blood-forming organs (B) | 2 |  |  |  |
| Cardiovascular system (C) | 22 |  |  |  |
| Dermatologicals (D) | 6 |  |  |  |
| Genitourinary system and sex hormones (G) | 4 |  |  |  |
| Anti-infectives for systemic use (J) | 1 |  |  |  |
| Antineoplastic and immunomodulating agents (L) | 3 |  |  |  |
| Musculoskeletal system (M) | 7 |  |  |  |
| Nervous system (N) | 30 |  |  |  |
| Respiratory system (R) | 2 |  |  |  |
| Sensory organs (S) | 1 |  |  |  |
| Various (V) | 2 |  |  |  |
| Total | 101 |  |  |  |
