# Supplementary material for: Standard-based comprehensive detection of adverse drug reaction signals from nursing statements and laboratory results in electronic health records
Source: J Am Med Inform Assoc. 2017 Jan 13;24(4):697–708. doi: 10.1093/jamia/ocw168 (PMC7651894; doi:10.1093/jamia/ocw168)
Supplement: Supplementary Data [file ocw168_supp.zip › Supplementary_Table_S2_b_r.docx]

| **Supplementary Table S2** 101 precautionary study drug list **(b)** Drugs are mapped to product name used at SNUH (*n*=672) | | |
| --- | --- | --- |
|  |  |  |
| **Drug name** | **Product name used at SNUH (n=672)** |  |
| aceclofenac | Aceclofenac 100mg |  |
| aceclofenac | Aceclofenac 100mgCap |  |
| aceclofenac | Aceclofenac 70mg cap |  |
| aceclofenac | Aceclofenac 100mg |  |
| aceclofenac | Aceclofenac 100mg |  |
| aceclofenac | Aceclofenac 100mgCap |  |
| aceclofenac | Aceclofenac 70mg cap |  |
| acetaminophen | Perfalgan 1g/100ml btl(Acetaminophen) |  |
| acetaminophen | Tylenol ER 650mg tab (Acetaminophen) |  |
| acetaminophen | Tylenol 80mg tab (Acetaminophen) |  |
| acetaminophen | Tylenol suspension 32mg/ml(Acetaminophen) |  |
| acetaminophen | Rhinapen 3.2% syr (Acetaminophen) |  |
| acetaminophen | Setopen 3.2%syrup(Acetaminophen) |  |
| acetaminophen | Geworin-i chewable tab.(Acetaminophen) |  |
| acetaminophen | Suspen suppository 125mg (Acetaminophen) |  |
| acetaminophen | Endapen 300mg tab (Acetaminophen) |  |
| acetaminophen | Acetaminophen375 Che |  |
| acetaminophen | Tylenol ER 650mg tab (Acetaminophen) |  |
| acetaminophen | Geworin-i chewable tab.(Acetaminophen) |  |
| acetaminophen | Perfalgan 1g/100ml btl(Acetaminophen) |  |
| acetaminophen | Suspen suppository 125mg (Acetaminophen) |  |
| acetaminophen | Rhinapen 3.2% syr (Acetaminophen) |  |
| acetaminophen | Acetaminophen 500mg |  |
| acetaminophen | Ketotop-p Acetaminophen 650mg |  |
| acetaminophen | SAMe acetaminophen 650mg |  |
| acetaminophen | RA Acetaminophen 500mg tab |  |
| alprazolam | Zanapam 1mg Tab(Alprazolam) |  |
| alprazolam | Alpram 0.5mg (Alprazolam) |  |
| alprazolam | Xanax 0.25mg tab(Alprazolam) |  |
| alprazolam | Alprazolam 0.25mg |  |
| alprazolam | Alprazolam 0.5mg |  |
| alprazolam | Zanapam 1mg Tab(Alprazolam) |  |
| alprazolam | Alpram 0.25mg (Alprazolam) |  |
| alprazolam | Alpram 0.5mg (Alprazolam) |  |
| amiodarone | Cordarone 200mg tab(Amiodarone) |  |
| amiodarone | Cordarone 150mg inj (Amiodarone) |  |
| amiodarone | Cordarone 200mg tab(Amiodarone) |  |
| amiodarone | Cordarone 150mg inj (Amiodarone) |  |
| amitriptyline | Etravil 25mg tab (Amitriptyline) |  |
| amitriptyline | Etravil 10mg tab (Amitriptyline) |  |
| amitriptyline | Etravil 25mg tab (Amitriptyline) |  |
| amitriptyline | Amitriptyline 5mg |  |
| amlodipine | Amlostar 5mg tab(Amlodipine adipate) |  |
| amlodipine | Amlostar 5mg tab(Amlodipine adipate) |  |
| amlodipine | Levotension 2.5mg tab(S-amlodipine besylate) |  |
| amlodipine | Levotension 2.5mg tab(S-amlodipine besylate) |  |
| amlodipine | Amlodipine orot. 5mg |  |
| amlodipine | Amlodipine 5mg |  |
| amlodipine | Amlodipine 5mg |  |
| amlodipine | Amlodipine 5mg |  |
| amlodipine | S-Amlodipine 2.5mg |  |
| amlodipine | Amlodipine 5mg |  |
| amlodipine | Amlodipine Camsylate |  |
| amlodipine | Amlodipine 5mg |  |
| amlodipine | Amlodipine 5mg |  |
| amlodipine | Amlodipine (STUDY) |  |
| amlodipine | Amlodipine 5mg |  |
| amlodipine | Amlodipine 5mg |  |
| amlodipine | Amlodipine Camsylate |  |
| amlodipine | Amlodipine orot. 5mg |  |
| amlodipine | Amlodipine(STUDY) |  |
| amlodipine | (Study)068Amlodipine |  |
| amlodipine | Amlodipine (STUDY) |  |
| amlodipine | Lodien 2.5mg tab(S-amlodipine nicotinate) |  |
| amlodipine | Lodien 2.5mg tab(S-amlodipine nicotinate) |  |
| amphotericin | Fungizone* 50mg (Amphotericin-B) |  |
| amphotericin | Ambisome 50mg vial(Amphotericin B liposoma) |  |
| aprepitant | Aprepitant 80mg |  |
| aprepitant | Aprepitant 125mg |  |
| aprepitant | Aprepitant 80mg |  |
| aprepitant | Aprepitant 125mg |  |
| aspirin | Rhonal 500mg tab (Aspirin microcoated) |  |
| aspirin | Rhonal for children 100mg tab(Aspirin microcoated) |  |
| aspirin | Arthalgyl 90% inj.(Aspirin lysinate) |  |
| aspirin | Astrix 100mg cap (Aspirin enteric coated) |  |
| aspirin | Praspirin 300mg cap(Triflusal) |  |
| aspirin | Aspirin protect 100mg(Aspirin enteric coated) |  |
| aspirin | Aspirin pow |  |
| aspirin | Aspirin 10mg |  |
| aspirin | Aspirin 100mg |  |
| aspirin | Aspirin 500mg |  |
| aspirin | Astrix 100mg cap (Aspirin enteric coated) |  |
| aspirin | Arthalgyl 90% inj.(Aspirin lysinate) |  |
| aspirin | Aspirin micro 100mg |  |
| aspirin | Rhonal 500mg tab (Aspirin microcoated) |  |
| aspirin | Aspirin protect 100mg(Aspirin enteric coated) |  |
| aspirin | Aspirin325mg(STUDY) |  |
| aspirin | Aspirin100 (STUDY) |  |
| atenolol | Atenolol 50mg |  |
| atenolol | Atenolol 25mg |  |
| atenolol | Tenormin 25mg tab(Atenolol) |  |
| atenolol | Tenormin 50mg tab (Atenolol) |  |
| atropine | Atropine 0.5mg |  |
| atropine | Atropine 10mg/20ml |  |
| atropine | Atropine 0.5mg |  |
| atropine | Atropine oph 1% 15ml |  |
| atropine | Atropine pow |  |
| atropine | Atropine 10mg/20ml |  |
| atropine | Atropine oph 1% 3.5g |  |
| atropine | Atropine oph 1% 15ml |  |
| atropine | Atropine pow X1000(* |  |
| atropine | Atropine 0.5mg |  |
| benztropine | Benztropine 2mg |  |
| benztropine | Benztropine 2mg tab(Benztropine) |  |
| bisacodyl | Bisacodyl 5mg Tab |  |
| bisacodyl | Bisacodyl 10mg Supp |  |
| bisacodyl | Bisacodyl 5mg tab신일 (Bisacodyl) |  |
| bisacodyl | Dulcolax Supp (Bisacodyl) |  |
| bisacodyl | Bisacodyl(STUDY) |  |
| bisoprolol | Bisoprolol 5mg tab |  |
| bisoprolol | Bisoprolol 5mg tab |  |
| bisoprolol | Bisoprolol 2.5mg tab |  |
| bisoprolol | Bisoprolol 5mg tab |  |
| bisoprolol | Bisoprolol 2.5mg tab |  |
| budesonide | Budecort 0.5mg/2ml amp(Micronized Budesonide) |  |
| budesonide | Entocort rectal set(Budesonide) |  |
| budesonide | Pulmicort Respules 0.5mg/2ml amp(Budesonide) |  |
| budesonide | Pulmicort Turbuhaler 200mcg 100dose(Budesonide) |  |
| budesonide | BudesonideTurb100dos |  |
| budesonide | Pulmicort Turbuhaler 200mcg 100dose(Budesonide) |  |
| budesonide | Budesonide nasal tur |  |
| budesonide | Budesonide nasal sol |  |
| budesonide | Entocort rectal set(Budesonide) |  |
| budesonide | Budecort 0.5mg/2ml amp(Micronized Budesonide) |  |
| bupropion | Wellbutrin SR 300mg tab(Bupropion) |  |
| bupropion | Wellbutrin XL 300mg tab(Bupropion) |  |
| bupropion | Wellbutrin SR 150mg tab(Bupropion) |  |
| bupropion | Bupropion PET(STUDY) |  |
| bupropion | Bupropion PET(STUDY) |  |
| capsaicin | Capsaicin 0.025% 20g |  |
| capsaicin | Capsaicin 0.075% 20g |  |
| capsaicin | Capsaicin 0.025%56.6 |  |
| capsaicin | Capsaicin 0.025% 20g |  |
| capsaicin | Capsaicin 0.075%56.6 |  |
| capsaicin | Capsaicin 0.075% 20g |  |
| carvedilol | Carvedilol 25mg |  |
| carvedilol | Carvedilol 12.5mg |  |
| carvedilol | Carvedilol 6.25mg |  |
| carvedilol | Dilatrend 12.5mg tab (Carvedilol) |  |
| carvedilol | Dilatrend 12.5mg tab (Carvedilol) |  |
| carvedilol | Dilatrend 25mg tab (Carvedilol) |  |
| chlordiazepoxide | Liberty 5mg tab (Chlordiazepoxide) |  |
| chlordiazepoxide | Chlordiazepoxide10mg |  |
| chlordiazepoxide | Liberty 5mg tab (Chlordiazepoxide) |  |
| chlordiazepoxide | Chlordiazepoxide pow |  |
| chlordiazepoxide | Liberty 5mg tab (Chlordiazepoxide) |  |
| chlorpheniramine | Chlorpheniramine 2mg |  |
| chlorpheniramine | Chlorpheniramine 2mg |  |
| chlorpheniramine | Chlorpheniramine |  |
| chlorpheniramine | Chlorpheniramine |  |
| chlorpromazine | Chlopromazine 100mg tab (Chlorpromazine) |  |
| chlorpromazine | Chlopromazine 50mg tab (Chlorpromazine) |  |
| chlorpromazine | Chlorpromazine 200mg tab(Chlorpromazine) |  |
| chlorpromazine | Chlopromazine 100mg tab (Chlorpromazine) |  |
| chlorpromazine | Chlorpromazine 12.5m |  |
| chlorpromazine | Chlorpromazine 200mg tab(Chlorpromazine) |  |
| chlorpromazine | Chlorpromazine 25mg |  |
| chlorpromazine | Chlorpromazine 50mg |  |
| chlorpromazine | Chlorpromazine 25mg |  |
| chlorpromazine | Chlorpromazine 50mg |  |
| cimetidine | Cimet tab 200mg(Cimetidine) |  |
| cimetidine | Himetin 400mg tab (Cimetidine) |  |
| cimetidine | Cimet tab 200mg(Cimetidine) |  |
| cimetidine | Himetin 400mg tab (Cimetidine) |  |
| ciprofloxacin | Cycin 250mg (Ciprofloxacin) |  |
| ciprofloxacin | Ciplus 250mg tab |  |
| ciprofloxacin | Citopcin 100mg bag (Ciprofloxacin) |  |
| ciprofloxacin | Citopcin 200mg bag (Ciprofloxacin) |  |
| ciprofloxacin | Citopcin 400mg bag(Ciprofloxacin) |  |
| citalopram | Citalopram 20mg |  |
| citalopram | Lexapro 10mg tab(Escitalopram oxalate) |  |
| citalopram | Escitalopram (STUDY) |  |
| clopidogrel | Cloart 75mg tab(clopidogrel) |  |
| clopidogrel | Clopidogrel 75mg KOR |  |
| clopidogrel | Clopidogrel 75mg KOR |  |
| clopidogrel | Plavix 75mg (Clopidogrel) |  |
| clopidogrel | Plavix 75mg tab(Clopidogrel) |  |
| clopidogrel | Clopidogrel(study) |  |
| clopidogrel | Clopidogrel 75mg |  |
| clopidogrel | Clopidogrel (STUDY) |  |
| clopidogrel | Clopidogrel 75mg |  |
| clopidogrel | Clopidogrel bisulfate 75mg tab |  |
| clopidogrel | Clopidogrel bisulfate 75mg tab |  |
| clozapine | Clozaril 100mg tab (Clozapine) |  |
| clozapine | Clozaril 25mg tab(Clozapine) |  |
| clozapine | Clozaril 100mg tab (Clozapine) |  |
| clozapine | Clozaril 25mg tab(Clozapine) |  |
| diazepam | Diazepam 2mg tab (Diazepam) |  |
| diazepam | Diazepam 5mg |  |
| diazepam | Diazepam 10mg |  |
| diazepam | Diazepam 2mg |  |
| diazepam | Diazepam 5mg tab (Diazepam) |  |
| diazepam | Diazepam10mg,2ml (Diazepam) |  |
| diclofenac | Dicloflex plaster(Diclofenac) |  |
| diclofenac | Rheumastop plaster 120mg (Diclofenac) |  |
| diclofenac | Diclan eyedrops 0.1% 5ml (Diclofenac) |  |
| diclofenac | Rheumastop plaster 120mg (Diclofenac) |  |
| diclofenac | Diclan eyedrops 0.1% 5ml (Diclofenac) |  |
| diclofenac | Diclofenac 75mg |  |
| diclofenac | Diclofenac 100mg |  |
| diclofenac | Diclofenac 1% 30g |  |
| digoxin | Digosin 0.25mg tab (Digoxin) |  |
| digoxin | Digoxin 0.25mg |  |
| digoxin | Digoxin sy 0.05mg/ml |  |
| digoxin | Digoxin(study) |  |
| digoxin | Digoxin 0.02mg |  |
| digoxin | Digosin 0.25mg tab (Digoxin) |  |
| digoxin | Digoxin 0.25mg inj (Digoxin) |  |
| digoxin | Cadef 0.05mg/ml elx. (Digoxin) |  |
| digoxin | Digoxin pow X10000(* |  |
| diltiazem | Diltiazem 30mg |  |
| diltiazem | Diltiazem 90mg |  |
| diltiazem | Diltiazem 50mg |  |
| diltiazem | Diltiazem 30mg |  |
| diltiazem | Diltiazem 90mg |  |
| diltiazem | Diltiazem 50mg |  |
| dolasetron | Dolasetron 200mg |  |
| dolasetron | Dolasetron 200mg |  |
| dolasetron | Dolasetron 100mg/5ml |  |
| dolasetron | Dolasetron 12.5mg |  |
| dolasetron | Dolasetron 12.5mg |  |
| dolasetron | Dolasetron 100mg/5ml |  |
| dolasetron | Dolasetron 200mg |  |
| dolasetron | Dolasetron 100mg/5ml |  |
| doxazosin | Pzocin XL 4mg tab(Doxazosin) |  |
| doxazosin | Doxazon SL C.T. 4mg tab(Doxazosin) |  |
| doxazosin | Cardura XL 4mg tab(Doxazosin) |  |
| doxazosin | Doxazosin 2mg |  |
| doxazosin | Doxazosin 2mg |  |
| doxazosin | Carzosin 2mg tab(doxazosin) |  |
| doxazosin | Doxazon SL C.T. 4mg tab(Doxazosin) |  |
| doxycycline | Unidoxy cap 100mg (doxycycline) |  |
| doxycycline | Doxycycline 100mg |  |
| doxycycline | Unidoxy cap 100mg (doxycycline) |  |
| enalapril | Lenipril 10mg tab (Enalapril) |  |
| enalapril | Enaprin 5mg tab(Enalapril) |  |
| epinephrine | Epinephrine 1mg |  |
| epinephrine | Epinephrine Kit |  |
| epinephrine | Epinephrine Jr Kit |  |
| epinephrine | Epinephrine sol 50ml |  |
| epinephrine | Epinephrine Jr Kit |  |
| epinephrine | Epinephrine Kit |  |
| epinephrine | Epinephrine 1mg |  |
| epinephrine | Epinephrine sol 50ml |  |
| ergoloid | Ergoloid mesylat 1mg |  |
| ergoloid | Ergoloid mesyl 15mg |  |
| esmolol | Esmolol 100mg |  |
| esmolol | Esmolol 2.5g |  |
| esmolol | Esmolol 100mg |  |
| esmolol | Esmolol 2.5g |  |
| etodolac | Lodine 600mg SR tab(Etodolac) |  |
| etodolac | Etodolac 200mg cap |  |
| etodolac | Lodine 200mg cap(Etodolac) |  |
| etodolac | Lodine 600mg SR tab(Etodolac) |  |
| etoposide | E.P.S 100mg inj (Etoposide) |  |
| etoposide | Lastet 25mg cap (Etoposide) |  |
| famotidine | Famotidine 20mg |  |
| famotidine | Famotidine 20mg Disp |  |
| famotidine | Famotidine 20mg tab |  |
| famotidine | Famotidine 20mg |  |
| famotidine | Famotidine 20mg tab |  |
| famotidine | Famotidine 40mg tab |  |
| famotidine | Famotidine 20mg Disp |  |
| famotidine | Famotidine 20mg |  |
| famotidine | Famotidine (Study) |  |
| flecainide | Tambacor 50mg tab(Flecainide) |  |
| flecainide | Flecainide 50mg |  |
| flecainide | Flecainide 50mg |  |
| flecainide | Tambacor 50mg tab(Flecainide) |  |
| flecainide | Flecainide(study) |  |
| fluconazole | Plunazol 50mg cap (Fluconazole) |  |
| fluconazole | Oneflu 100mg inj. (Fluconazole) |  |
| flumazenil | Flumazenil 0.5mg |  |
| flumazenil | Flumazenil 0.3mg |  |
| flumazenil | Flumazenil 0.5mg |  |
| flumazenil | Flumazenil 0.3mg |  |
| fluorouracil | Fluorouracil-5 500mg inj (Fluorouracil) |  |
| fluorouracil | Fluorouracil-5 250mg inj (Fluorouracil) |  |
| fluoxetine | Prozac 20mg Cap (Fluoxetine) |  |
| fluoxetine | Fluoxetine 10mg cap |  |
| fluoxetine | Fluoxetine 10mg cap |  |
| fluoxetine | Prozac 20mg Cap (Fluoxetine) |  |
| fluoxetine | Prozac 20mg Cap (Fluoxetine) |  |
| fluoxetine | Prozac dispersible tab 20mg(Fluoxetine) |  |
| fluticasone | Fluticasone nasal |  |
| fluticasone | Fluticasone nasal |  |
| fluticasone | Fluticasone disk 100 |  |
| fluticasone | Fluticasone inh 250m |  |
| fluticasone | Flixonase Nasal Spray 120dos (Fluticasone) |  |
| fluticasone | Flixotide evohaler 250mcg/120dos(Fluticasone) |  |
| fluticasone | Flixotide Diskus 250mcg/60dos (Fluticasone) |  |
| fluticasone | Cutivate Cream 0.05% 10g(Fluticasone) |  |
| fluticasone | Flixotide Diskus 250mcg/60dos (Fluticasone) |  |
| fluticasone | Flixotide evohaler 250mcg/120dos(Fluticasone) |  |
| fluticasone | Cutivate Cream 0.05% 10g(Fluticasone) |  |
| fluticasone | Flixotide nebule 2mg/2ml plastic amp(Fluticasone) |  |
| gentamicin | Gentacin 20mg inj (Gentamicin) |  |
| gentamicin | Gentamicin 80mg inj (Gentamicin) |  |
| haloperidol | Haloperidol 5mg (Haloperidol) |  |
| haloperidol | Peridol 5mg tab (Haloperidol) |  |
| haloperidol | Haloperidol 1.5mg |  |
| haloperidol | Haloperidol 10mg |  |
| haloperidol | Haloperidol 3mg |  |
| haloperidol | Haloperidol 5mg |  |
| haloperidol | Haloperidol 0.5mg |  |
| haloperidol | Peridol 10mg tab (Haloperidol) |  |
| haloperidol | Haloperidol 1.5mg |  |
| haloperidol | Peridol 3mg tab (Haloperidol) |  |
| haloperidol | Haloperidol 5mg |  |
| haloperidol | Haloperidol 50mg |  |
| haloperidol | Haloperidol 5mg (Haloperidol) |  |
| hydroxyzine | Hydroxyzine 10mg |  |
| hydroxyzine | Hydroxyzine sy 2mg/m |  |
| hydroxyzine | Hydroxyzine 10mg |  |
| hydroxyzine | Hydroxyzine 10mg |  |
| hydroxyzine | Hydroxyzine 10mg |  |
| hydroxyzine | Hydroxyzine sy 2mg/m |  |
| ibuprofen | Carol syr 20mg/ml (Ibuprofen) |  |
| ibuprofen | Dexibuprofen sy |  |
| ibuprofen | Carol syr 20mg/ml (Ibuprofen) |  |
| ibuprofen | Ibuprofen 400mg |  |
| indomethacin | Indocin* 1mg inj(Indomethacin) |  |
| indomethacin | Indocin* 1mg inj(Indomethacin) |  |
| indomethacin | Indomethacin SR 25mg |  |
| indomethacin | Indomethacin 25mg |  |
| indomethacin | Indocin* 1mg inj(Indomethacin) |  |
| indomethacin | Indomethacin SR 25mg |  |
| indomethacin | Indomethacin25mg |  |
| ketoprofen | Ketoprofen 100mg inj (Ketoprofen) |  |
| ketoprofen | Topren 100mg inj (Ketoprofen) |  |
| ketoprofen | Ketotop 30mg/g 50g gel(Ketoprofen) |  |
| ketoprofen | Rheuma RX plaster 30mg patch(Ketoprofen) |  |
| ketoprofen | Ketoprofen 100mg |  |
| ketoprofen | Ketoprofen 30mg/pat |  |
| ketoprofen | Rheuma RX plaster 30mg patch(Ketoprofen) |  |
| ketoprofen | Ketotop 30mg/g 50g gel(Ketoprofen) |  |
| ketoprofen | Topren 100mg inj (Ketoprofen) |  |
| ketorolac | Ketro 10mg tab(ketorolac) |  |
| ketorolac | Ketoracin 10mg tab(Ketorolac) |  |
| ketorolac | Trolac 30mg inj (Ketorolac) |  |
| ketorolac | Acular oph soln 0.5% 5ml(Ketorolac) |  |
| ketorolac | Ketoracin 10mg tab(Ketorolac) |  |
| ketorolac | Acular oph soln 0.5% 5ml(Ketorolac) |  |
| ketorolac | Trolac 30mg inj (Ketorolac) |  |
| labetalol | Labetalol 100mg |  |
| labetalol | Labetalol 100mg |  |
| labetalol | Labetalol 100mg |  |
| labetalol | Labetalol 100mg |  |
| lansoprazole | Lansoprazole 30mg |  |
| lansoprazole | Lansoprazole 15mg |  |
| lansoprazole | LansoprazoleLFDT30mg |  |
| lansoprazole | Lansoprazole 30mg |  |
| lansoprazole | Lansoprazole 15mg |  |
| lansoprazole | LansoprazoleLFDT30mg |  |
| lansoprazole | Lansoprazole(Study) |  |
| lansoprazole | (Study) Lansoprazole |  |
| loperamide | Loperamide 2mg cap |  |
| loperamide | Loperamide 2mg cap (Loperamide) |  |
| loperamide | LOPERAMIDE(STUDY) |  |
| loperamide | Loperamide 2mg cap (Loperamide) |  |
| loperamide | Loperamide 2mg |  |
| lorazepam | Ativan 4mg inj (Lorazepam) |  |
| lorazepam | Ativan 0.5mg tab (Lorazepam) |  |
| lorazepam | Ativan 1mg tab (Lorazepam) |  |
| lorazepam | Loravan 0.5mg tab (Lorazepam) |  |
| lorazepam | Loravan 1mg tab (Lorazepam) |  |
| lorazepam | Loravan 0.5mg tab (Lorazepam) |  |
| lorazepam | Loravan 1mg tab (Lorazepam) |  |
| lorazepam | Lorazepam 1mg tab |  |
| meloxicam | Meloxicam 7.5mg cap |  |
| meloxicam | Meloxicam 15mg cap |  |
| meloxicam | Meloxicam 7.5mg cap |  |
| meloxicam | Meloxicam 15mg cap |  |
| meloxicam | Meloxicam 15mg cap |  |
| meloxicam | Mobic 7.5mg cap (Meloxicam) |  |
| meloxicam | Mobic 15mg cap (Meloxicam) |  |
| methotrexate | MTX 2.5mg tab (Methotrexate) |  |
| methotrexate | Methotrexate 50mg inj (Methotrexate) |  |
| methotrexate | Methotrexate 50mg inj (Methotrexate) |  |
| methylphenidate | Concerta OROS tab 27mg(methylphenidate) |  |
| methylphenidate | Penid 10mg tab (Methylphenidate) |  |
| methylphenidate | Concerta OROS tab 18mg(methylphenidate) |  |
| methylphenidate | Concerta OROS tab 27mg(methylphenidate) |  |
| methylphenidate | Penid 10mg tab (Methylphenidate) |  |
| methylphenidate | Concerta OROS tab 18mg(methylphenidate) |  |
| methylphenidate | Metadate CD 20mg Cap (Methylphenidate) |  |
| metoclopramide | Macperan 10mg inj (Metoclopramide) |  |
| metoclopramide | Meckool inj 10mg/2ml amp(Metoclopramide) |  |
| metoclopramide | Macperan 10mg inj (Metoclopramide) |  |
| metoclopramide | Meckool inj 10mg/2ml amp(Metoclopramide) |  |
| metoclopramide | Metoclopramide N |  |
| metoprolol | Metoprolol ER 95mg |  |
| metoprolol | Metoprolol 100mg |  |
| metoprolol | Metoprolol 100mg |  |
| metoprolol | Metoprolol ER 95mg |  |
| mirtazapine | Remeron SolTab 30mg(Mirtazapine) |  |
| mirtazapine | Mirtazapine 30mg tab |  |
| mirtazapine | Remeron 15mg tab(Mirtazapine) |  |
| mirtazapine | Remeron SolTab 30mg(Mirtazapine) |  |
| mometasone | Nasonex* nasal 140dose (Mometasone furoate) |  |
| mometasone | Nasonex* nasal 140dose (Mometasone furoate) |  |
| naloxone | Naloxone 2mg |  |
| naloxone | Naloxone 0.4mg |  |
| naloxone | Naloxone 2mg |  |
| naloxone | Naloxone 0.4mg |  |
| naproxen | Naxen-F 500mg tab (Naproxen) |  |
| naproxen | Naxen-F 500mg tab (Naproxen) |  |
| nicardipine | Nicardipine SR 40mg |  |
| nicardipine | Nicardipine 10mginj. |  |
| nicardipine | Nicardipine 20mg |  |
| nicardipine | Nicardipine 20mg |  |
| nicardipine | Nicardipine SR 40mg |  |
| nicardipine | Nicardipine 10mginj. |  |
| nifedipine | Nifedipine 10mg cap (Nifedepine) |  |
| nifedipine | Adalat OROS 60mg tab (Nifedipine) |  |
| nifedipine | Nifedipine 5mg |  |
| nifedipine | Nifedipine 20mg tab |  |
| nifedipine | Nifedipine 30mg tab |  |
| nifedipine | Nifedipine 10mg cap (Nifedepine) |  |
| nifedipine | Nifedipine 20mg tab |  |
| nifedipine | Adalat* OROS 30mg tab (Nifedipine) |  |
| nifedipine | Nifedipine 5mg cap(Nifedipine) |  |
| nifedipine | Adalat OROS 60mg tab (Nifedipine) |  |
| nifedipine | Nifedipine 10mg |  |
| nifedipine | (Study)068Nifedipine |  |
| nortriptyline | Nortriptyline 10mg |  |
| nortriptyline | Nortriptyline 25mg |  |
| nortriptyline | Nortriptyline 10mg |  |
| nortriptyline | Nortriptyline 25mg |  |
| nortriptyline | Nortriptyline(STUDY) |  |
| olanzapine | Zyprexa 5mg tab (Olanzapine) |  |
| olanzapine | Zyprexa 5mg tab (Olanzapine) |  |
| olanzapine | Zyprexa 2.5mg tab(Olanzapine) |  |
| olanzapine | Zyprexa Intra Muscular 10mg(Olanzapine) |  |
| olanzapine | Olanzapine 10mg tab |  |
| olanzapine | Olanzapine(STUDY) |  |
| olanzapine | Zyprexa 10mg tab (Olanzapine) |  |
| olanzapine | Zyprexa 2.5mg tab(Olanzapine) |  |
| olanzapine | Zyprexa 5mg tab (Olanzapine) |  |
| olanzapine | Olanzapine 7.5mg tab |  |
| olanzapine | Olanzapine(STUDY) |  |
| olanzapine | Olanzapine(Study) |  |
| olanzapine | Zyprexa 10mg tab (Olanzapine) |  |
| olanzapine | Zyprexa 5mg tab (Olanzapine) |  |
| ondansetron | Ondansetron 8mg |  |
| ondansetron | Ondansetron 8mg |  |
| ondansetron | Ondansetron 4mg |  |
| ondansetron | Ondansetron 8mg |  |
| ondansetron | Ondansetron s.l. 4mg |  |
| ondansetron | Ondansetron s.l. 8mg |  |
| ondansetron | Ondansetron 4mg |  |
| ondansetron | Ondansetron 4mg |  |
| ondansetron | Ondansetron 8mg |  |
| ondansetron | Ondansetron 8mg |  |
| ondansetron | Ondansetron s.l. 4mg |  |
| ondansetron | Ondansetron s.l. 8mg |  |
| ondansetron | Ondansetron(study) |  |
| oseltamivir | Tamiflu 75mg cap(Oseltamivir) |  |
| oseltamivir | Oseltamivir 75mg |  |
| oxybutynin | Obutin 5mg tab (Oxybutynin) |  |
| oxybutynin | Obutin 5mg tab (Oxybutynin) |  |
| oxybutynin | Lyrinel OROS 10mg tab (Oxybutynin) |  |
| oxybutynin | Lyrinel OROS 5mg tab (Oxybutynin) |  |
| oxybutynin | Oxybutynin solution |  |
| oxybutynin | Obutin 5mg tab (Oxybutynin) |  |
| oxybutynin | Lyrinel OROS 10mg tab (Oxybutynin) |  |
| oxybutynin | Oxybutynin irr.sol(* |  |
| oxybutynin | Oxybutynin pow X100 |  |
| oxybutynin | Lyrinel OROS 5mg tab (Oxybutynin) |  |
| paliperidone | Paliperidone 3mg |  |
| paliperidone | Paliperidone 6mg |  |
| paliperidone | Paliperidone 9mg |  |
| paliperidone | Paliperidone 3mg |  |
| paliperidone | Paliperidone 6mg |  |
| paliperidone | Paliperidone 9mg |  |
| paliperidone | PSY1008 Paliperidone 150mg |  |
| paliperidone | PALIPERIDONE(STUDY) |  |
| paliperidone | PSY1008 Paliperidone 100mg |  |
| paliperidone | Paliperidone ER 6mg |  |
| paliperidone | Paliperidone(STUDY) |  |
| paliperidone | PSZ-3002 Paliperidone ER |  |
| palonosetron | Palonosetron 0.25mg |  |
| palonosetron | Palonosetron (STUDY) |  |
| palonosetron | Palonosetron 0.25mg |  |
| paroxetine | Seroxate 20mg tab (Paroxetine) |  |
| paroxetine | Paroxetine CR 12.5mg |  |
| paroxetine | Paroxetine CR 12.5mg |  |
| paroxetine | Paroxetine(Study) |  |
| phenylephrine | Phenylephrine inj. 1% 1ml |  |
| phenylephrine | Mydfrin 2.5% 5ml oph (Phenylephrine) |  |
| phenylephrine | Phenylephrine HCl Eye Drops Geyer 10% |  |
| phenylephrine | Phenylephrine 10mg |  |
| phenylephrine | Mydfrin 2.5% 5ml oph (Phenylephrine) |  |
| phenylephrine | Phenylephrine inj. 1% 1ml |  |
| piroxicam | Trast patch (Piroxicam) |  |
| piroxicam | Trast gel 0.5% 50g(Piroxicam) |  |
| piroxicam | Trast patch (Piroxicam) |  |
| piroxicam | Brexin 10mg tab(Piroxicam b cyclodextrin) |  |
| piroxicam | Murupe patch(Piroxicam) |  |
| piroxicam | Brexin 10mg tab(Piroxicam b cyclodextrin) |  |
| piroxicam | Trast patch (Piroxicam) |  |
| piroxicam | Trast gel 0.5% 50g(Piroxicam) |  |
| piroxicam | Piroxicam 20mg |  |
| piroxicam | Piroxicam (STUDY) |  |
| prazosin | Minecin 1mg tab (Prazosin) |  |
| prazosin | Prazosin 2mg |  |
| prazosin | Prazosin 2mg |  |
| prednisolone | Prednisolone 1mg/ml |  |
| prednisolone | Prednisolone pow X40 |  |
| prednisolone | Prednisolone pow |  |
| prednisolone | Prednisolone 1mg/ml |  |
| prednisolone | Prednisolone(study) |  |
| propranolol | Pranol 40mg tab(Propranolol) |  |
| propranolol | Indenol 10mg tab (Propranolol) |  |
| propranolol | Indenol 10mg tab (Propranolol) |  |
| propranolol | Pranol 40mg tab(Propranolol) |  |
| propranolol | Inderal LA 80mg cap (Propranolol) |  |
| quetiapine | Seroquel 200mg tab(Quetiapine) |  |
| quetiapine | Seroquel 200mg tab(Quetiapine) |  |
| quetiapine | Quetiapine 300mg XR |  |
| quetiapine | Quetiapine 400mg XR |  |
| quetiapine | Quetiapine 100mg tab |  |
| quetiapine | Seroquel 100mg tab(Quetiapine) |  |
| quetiapine | Seroquel 200mg tab(Quetiapine) |  |
| quetiapine | Seroquel 25mg tab(Quetiapine) |  |
| quetiapine | Quetiapine 300mg XR |  |
| quetiapine | Quetiapine 400mg XR |  |
| quetiapine | Ramosetron HCl 0.1mg |  |
| quetiapine | Ramosetron 0.3mg inj |  |
| quetiapine | Ramosetron HCl 0.1mg |  |
| ranitidine | Ranitidine 100mg |  |
| ranitidine | Ranitidine 50mg |  |
| ranitidine | Ranitidine 75mg tab |  |
| ranitidine | Ranitidine(STUDY) |  |
| ranitidine | Ranitidine 100mg |  |
| ranitidine | Ranitidine 75mg tab |  |
| ranitidine | Ranitidine bismuth |  |
| ranitidine | Ranitidine 50mg |  |
| ranitidine | Ranitidine(STUDY) |  |
| risperidone | Risperdal 0.5mg tab (Risperidone) |  |
| risperidone | Risperdal Quicklet 0.5mg tab(Risperidone) |  |
| risperidone | Risperdal Quicklet 1mg tab(Risperidone) |  |
| risperidone | Risperdal CONSTA 37.5mg inj.(Risperidone) |  |
| risperidone | Risperidone 1mg |  |
| risperidone | Risperidone 2mg |  |
| risperidone | Risperidone Disp2mg |  |
| risperidone | Risperidone 25mginj. |  |
| risperidone | Risperdal 0.5mg tab (Risperidone) |  |
| risperidone | Risperdal 1mg tab (Risperidone) |  |
| risperidone | Risperdal 2mg tab (Risperidone) |  |
| risperidone | Risperdal CONSTA 25mg inj.(Risperidone) |  |
| risperidone | Risperdal CONSTA 37.5mg inj.(Risperidone) |  |
| risperidone | Risperdal Quicklet 0.5mg tab(Risperidone) |  |
| risperidone | Risperdal Quicklet 1mg tab(Risperidone) |  |
| risperidone | Risperdal Quicklet 2mg tab(Risperidone) |  |
| risperidone | Risperidone 1mg |  |
| risperidone | Risperidone 2mg |  |
| rosuvastatin | Crestor 10mg tab (Rosuvastatin ) |  |
| rosuvastatin | Crestor 5mg tab (Rosuvastatin ) |  |
| rosuvastatin | Crestor 20mg tab (Rosuvastatin ) |  |
| sertraline | Zoloft 50mg tab (Sertraline) |  |
| sucralfate | Sucralfate 250mg tab |  |
| sucralfate | Sucralfate 15ml |  |
| sucralfate | Sucralfate 250mg tab |  |
| sulfasalazine | Sulfasalazine 500mg |  |
| sulfasalazine | Sulfasalazine 500mg |  |
| sulfasalazine | Sulfasalazine 500mg |  |
| sulfasalazine | Sulfasalazine(study) |  |
| sumatriptan | Imigran 50mg tab (Sumatriptan) |  |
| sumatriptan | Imigran 50mg tab (Sumatriptan) |  |
| sumatriptan | Imigran 50mg tab (Sumatriptan) |  |
| tacrolimus | Tacrobell 0.5mg cap(Tacrolimus) |  |
| tacrolimus | Tacrobell 1mg cap(Tacrolimus) |  |
| tacrolimus | Tacrobell 0.25mg cap(Tacrolimus) |  |
| tacrolimus | Protopic oint 0.1% 10g(Tacrolimus) |  |
| tacrolimus | Protopic oint 0.03% 10g(Tacrolimus) |  |
| tacrolimus | Prograf 1mg cap(Tacrolimus) |  |
| tacrolimus | Prograf 0.5mg cap(Tacrolimus) |  |
| tacrolimus | Prograf 5mg/1ml inj(Tacrolimus) |  |
| tacrolimus | Tacrobell 1mg cap(Tacrolimus) |  |
| tacrolimus | Tacrobell 0.25mg cap(Tacrolimus) |  |
| tacrolimus | Tacrobell 0.5mg cap(Tacrolimus) |  |
| tacrolimus | Prograf 1mg cap(Tacrolimus) |  |
| tacrolimus | Prograf 0.5mg cap(Tacrolimus) |  |
| tacrolimus | Prograf 5mg/1ml inj(Tacrolimus) |  |
| tacrolimus | Protopic oint 0.03% 10g(Tacrolimus) |  |
| tacrolimus | Protopic oint 0.1% 10g(Tacrolimus) |  |
| terazosin | Hytrin 5mg tab(Terazosin) |  |
| terazosin | TZC tab 2mg (Terazosin ) |  |
| terazosin | Hytrin 1mg tab (Terazosin) |  |
| terazosin | Hytrin 2mg tab (Terazosin) |  |
| terazosin | Hytrin 2mg tab (Terazosin) |  |
| terazosin | Terazocin 2mg |  |
| terazosin | Terazosin 2mg() |  |
| theophylline | Theoclear dry syr 200mg/g(Theophylline) |  |
| theophylline | Theoclear 130mg cap (Theophylline) |  |
| theophylline | Uniphyl SR 200mg tab (Theophylline QD) |  |
| theophylline | Uniphyl SR 400mg tab (Theophylline) |  |
| theophylline | Etheophyl 100mg (Theophylline) |  |
| theophylline | Etheophyl 200mg cap (Theophylline) |  |
| theophylline | Theophylline (S.R 100mg) |  |
| theophylline | Theoclear 130mg cap |  |
| theophylline | Theophylline (S.R 200mg) |  |
| theophylline | Uniphyl SR 200mg tab (Theophylline QD) |  |
| theophylline | Uniphyl SR 400mg tab (Theophylline) |  |
| ticlopidine | Clid 100mg tab (Ticlopidine) |  |
| ticlopidine | Clid 250mg tab (Ticlopidine) |  |
| ticlopidine | Clid 100mg tab (Ticlopidine) |  |
| tolterodine | tolterodine SR 4mg |  |
| tolterodine | Detrusitol SR 2mg cap(tolterodine) |  |
| tolterodine | Detrusitol SR 4mg cap (Tolterodine) |  |
| tolterodine | Detrusitol SR 4mg cap (Tolterodine) |  |
| tolterodine | Tolterodine 1mg tab |  |
| tolterodine | Tolterodine 2mg |  |
| tolterodine | Tolterodine 4mg |  |
| tolterodine | Detrusitol SR 2mg cap(tolterodine) |  |
| tolterodine | Detrusitol SR 4mg cap |  |
| tolterodine | Tolterodine |  |
| tolterodine | Tolterodine2mg |  |
| tolterodine | Tolterodine4mg |  |
| topiramate | Topamax 100mg tab (Topiramate) |  |
| topiramate | Topamax sprinkle cap 25mg(Topiramate) |  |
| topiramate | Topamax sprinkle cap 50mg(Topiramate) |  |
| topiramate | Topamax 100mg tab (Topiramate) |  |
| topiramate | Topiramate 25mg |  |
| topiramate | Topamax sprinkle cap 25mg(Topiramate) |  |
| topiramate | Topamax sprinkle cap 50mg(Topiramate) |  |
| topiramate | ATOM Topiramate |  |
| trazodone | Trazodone 25mg cap(Trazodone) |  |
| trazodone | Trazodone 25mg cap(Trazodone) |  |
| triamcinolone | Triamcinolone 200mg |  |
| triamcinolone | Triamcinolone 40mg |  |
| triamcinolone | Triamcinolone 20mg |  |
| triamcinolone | Triamcinolone 40mg |  |
| triamcinolone | Triamcinolone N 15mg |  |
| triamcinolone | Triamcinolone 200mg |  |
| triamcinolone | Triamcinolone 40mg |  |
| triamcinolone | Triamcinolone 20mg |  |
| triamcinolone | Triamcinolone N 15mg |  |
| triamcinolone | Triamcinolone 0.1%5g |  |
| triazolam | Triram 0.25mg tab (Triazolam) |  |
| triazolam | Triram 0.25mg tab (Triazolam) |  |
| venlafaxine | Venlafaxine37.5 XR |  |
| venlafaxine | Venlafaxine75 XR |  |
| venlafaxine | Venlafaxine 37.5mg |  |
| venlafaxine | Venlafaxine 37.5mg |  |
| venlafaxine | Venlafaxine37.5 XR |  |
| venlafaxine | Venlafaxine75 XR |  |
| verapamil | Verapamil 40mg |  |
| verapamil | Verapamil 40mg |  |
| verapamil | Verapamil SR 180mg |  |
| verapamil | Verapamil 5mg |  |
| verapamil | Verapamil 80mg |  |
| verapamil | Verapamil SR 180mg |  |
| verapamil | Verapamil 40mg |  |
| verapamil | Verapamil 80mg |  |
| verapamil | Verapamil 5mg |  |
| ziprasidone | Zeldox 20mg cap(Ziprasidone HCL) |  |
| ziprasidone | Zeldox 40mg cap(Ziprasidone HCL) |  |
| ziprasidone | Ziprasidone 80mg |  |
| ziprasidone | Ziprasidone 20mg |  |
| ziprasidone | Zeldox 20mg cap(Ziprasidone HCL) |  |
| ziprasidone | Zeldox 40mg cap(Ziprasidone HCL) |  |
| ziprasidone | Ziprasidone 80mg |  |
| zolpidem | Stilnox CR tab 12.5mg (Zolpidem) |  |
| zolpidem | Stilnox CR 6.25mg tab(Zolpidem) |  |
| zolpidem | Stilnox tab 10mg (Zolpidem) |  |
| zolpidem | Zolpilam 10mg tab(zolpidem) |  |
| zolpidem | Zolpid 10mg tab(Zolpidem) |  |
| zolpidem | Zolpilam 10mg tab(zolpidem) |  |
| zolpidem | Stilnox 12.5mg CR tab(Zolpidem) |  |
| zolpidem | Stilnox CR 6.25mg tab(Zolpidem) |  |
|  |  |  |
