# Supplementary material for: Standard-based comprehensive detection of adverse drug reaction signals from nursing statements and laboratory results in electronic health records
Source: J Am Med Inform Assoc. 2017 Jan 13;24(4):697–708. doi: 10.1093/jamia/ocw168 (PMC7651894; doi:10.1093/jamia/ocw168)
Supplement: Supplementary Data [file ocw168_supp.zip › Supplementary_Table_S3_a_r.docx]

| **Supplementary Table S3** Laboratory tests are mapped to SNUH codes. Laboratory test abnormality lists applied to CLEAR and MetaLAB. **(a)** Fifty-five laboratory tests and 156 laboratory test codes at SNUH applied to CLEAR and MetaLAB. | | | | |
| --- | --- | --- | --- | --- |
|  |  |  |  |  |
| **Used in CLEAR Algoritm** | **Used in MetaLAB Algoritm** | **Laboratory test name (*n*=55)** | **Laboratory test code at SNUH (*n*=156)** | **Laboratory test name at SNUH (*n*=156)** |
|  |  |  |  |  |
| O |  | Activated partial thromboplastin time | L2204 | aPTT |
| O |  | Activated partial thromboplastin time | L8041 | aPTT |
| O | O | Alanine transaminase | L0030 | GPT (ALT) [POCT] |
| O | O | Alanine transaminase | L3015 | GPT (ALT) |
| O | O | Alanine transaminase | L8153 | GPT (em) |
|  | O | Albumin | L3010 | Albumin |
|  | O | Albumin | L8163 | Albumin (em) |
| O | O | Alkaline phosphatase | L0028 | Alkaline phosphatase [POCT] |
| O | O | Alkaline phosphatase | L3012 | Alkaline phosphatase |
| O | O | Alkaline phosphatase | L8166 | Alkaline phosphatase (em) |
| O | O | Ammonia | L8142 | Ammonia (em) |
| O | O | Amylase | L0037 | Amylase [POCT] |
| O | O | Amylase | L3020 | Amylase(S) |
| O | O | Amylase | L31162 | Amylase (serum) |
| O | O | Amylase | L8139 | Amylase (serum, em) |
| O | O | Aspartate transaminase | L0029 | GOT (AST) [POCT] |
| O | O | Aspartate transaminase | L3014 | GOT (AST) |
| O | O | Aspartate transaminase | L8152 | GOT (em) |
| O | O | Basophil | L20128 | Basophil |
| O | O | Basophil | L80425 | Basophil |
| O | O | Blood urea nitrogen | L0023 | BUN [POCT] |
| O | O | Blood urea nitrogen | L3006 | BUN |
| O | O | Blood urea nitrogen | L8134 | BUN (serum, em) |
|  | O | Calcium | L3003 | Calcium |
|  | O | Calcium | L8160 | Calcium, total (em) |
|  | O | Calcium, ionized | L0010 | Calcium, ionized [POCT] |
|  | O | Calcium, ionized | L8133 | Calcium, ionized (em) |
|  | O | Chloride | L0008 | Chloride (serum, em) [POCT] |
|  | O | Chloride | L3045 | Chloride (serum) |
|  | O | Chloride | L8132 | Chloride (serum, em) |
| O | O | Cholesterol | L3008 | Cholesterol |
| O | O | Cholesterol | L8165 | Cholesterol (em) |
| O |  | Creatine kinase | L0012 | CKMB [POCT] |
| O |  | Creatine kinase | L0038 | CK (CPK) [POCT] |
| O |  | Creatine kinase | L3022 | CK (CPK) |
| O |  | Creatine kinase | L8156 | CK (em) |
| O |  | Creatine kinase | L8158 | CKMB (em) |
| O | O | Creatinine | L0031 | Creatinine [POCT] |
| O | O | Creatinine | L3041 | Creatinine |
| O | O | Creatinine | L31122 | Creatinine (serum) |
| O | O | Creatinine | L31242 | Creatinine (serum) |
| O | O | Creatinine | L31252 | Creatinine (serum) |
| O | O | Creatinine | L8135 | Creatinine |
|  | O | Creatinine Clearance | L31121 | Creatinine Clearance (24hrs urine, Ccr) |
|  | O | Creatinine Clearance | L31221 | KT/V Creatinine Clerarance (Ccr) (24hrs Urine) |
| O | O | Direct bilirubin | L3018 | Bilirubin, direct |
| O | O | Eosinophil | L2010 | Eosinophil count |
| O | O | Eosinophil | L20127 | Eosinophil |
| O | O | Eosinophil | L80424 | Eosinophil |
|  | O | ESR | L2008 | ESR |
| O | O | Fibrinogen | L2206 | Fibrinogen |
| O | O | Fibrinogen | L8045 | Fibrinogen (em) |
| O |  | Free thyroxine | L5202 | T4(free)(24) |
| O |  | Free thyroxine | L52021 | T4(free) |
| O |  | Free thyroxine | L7604 | T4(Total) |
| O |  | Free thyroxine | L76124 | T4(free) |
| O |  | Free thyroxine | L7624 | T4(free) |
| O | O | Gamma-glutamyl transpeptidase | L3016 | GGT |
| O | O | Gamma-glutamyl transpeptidase | L8167 | GGT (em) |
| O | O | Glucose | L0011 | Glucose [POCT] |
| O | O | Glucose | L0022 | Glucose [POCT] |
| O | O | Glucose | L3005 | Glucose |
| O | O | Glucose | L6106 | Glucose |
| O | O | Glucose | L8137 | Glucose (em) |
| O | O | Hematocrit | L0003 | Hct [POCT] |
| O | O | Hematocrit | L2004 | Hct |
| O | O | Hematocrit | L2022 | MCH |
| O | O | Hematocrit | L8003 | Hct (em) |
| O | O | Hematocrit | L8034 | MCH |
| O | O | Hemoglobin | L0002 | Hb [POCT] |
| O | O | Hemoglobin | L2003 | Hb |
| O | O | Hemoglobin | L2023 | MCHC |
| O | O | Hemoglobin | L6010 | Human Hb |
| O | O | Hemoglobin | L7910 | Human Hb |
| O | O | Hemoglobin | L8002 | Hb (em) |
| O | O | Hemoglobin | L8035 | MCHC |
| O | O | Hemoglobin | L81201 | Total Hb |
|  | O | Insulin | L7627 | Insulin |
|  | O | Iron | L3051 | Iron |
| O |  | Lactate dehydrogenase | L3023 | LD (LDH) |
| O |  | Lactate dehydrogenase | L8155 | LD (em) |
| O | O | LDL cholesterol | L3068 | LDL-Cholesterol |
| O | O | Lipase | L8157 | Lipase (em) |
| O | O | Lymphocyte | L20125 | Lymphocyte |
| O | O | Lymphocyte | L62062 | Lymphocyte |
| O | O | Lymphocyte | L80242 | Lymphocyte |
| O | O | Lymphocyte | L80422 | Lymphocyte |
|  | O | Magnesium | L3069 | Magnesium |
|  | O | Monocyte | L20126 | Monocyte |
|  | O | Monocyte | L80423 | Monocyte |
| O | O | Myoglobin | L0015 | Myoglobin [POCT] |
| O | O | Myoglobin | L7247 | Myoglobin |
| O | O | Myoglobin | L8194 | Myoglobin |
| O | O | Neutrophil | L20123 | Band neutrophil |
| O | O | Neutrophil | L20124 | Seg.neut. |
| O | O | Neutrophil | L80421 | Segmented neutrophil |
| O | O | Neutrophil | L804211 | Band neutrophil |
|  | O | pH | L00051 | pH [POCT] |
|  | O | pH | L6104 | pH |
|  | O | pH | L8008 | pH |
|  | O | pH | L81101 | pH |
|  | O | Phosphorus | L3004 | Phosphorus |
|  | O | Phosphorus | L31251 | Phosphorus (serum) |
|  | O | Phosphorus | L8161 | Phosphorus (em) |
| O | O | Platelet | L2009 | PLT |
| O | O | Platelet | L8031 | PLT (em) |
| O | O | Potassium | L0007 | Potassium (serum) [POCT] |
| O | O | Potassium | L0033 | Potassium (serum) [POCT] |
| O | O | Potassium | L3044 | Potassium (serum) |
| O | O | Potassium | L8131 | Potassium (serum, em) |
| O | O | Prolactin | L7617 | Prolactin |
| O | O | Prothrombin time | L22031 | PT (INR) |
| O | O | Prothrombin time | L22032 | PT (%) |
| O | O | Prothrombin time | L22033 | PT (sec) |
| O | O | Prothrombin time | L2279 | PT (Prothrombin time |
| O | O | Prothrombin time | L80441 | PT (INR) |
| O | O | Prothrombin time | L80442 | PT (%) |
| O | O | Prothrombin time | L80443 | PT (sec) |
| O | O | Red blood cell | L2002 | RBC |
| O | O | Red blood cell | L8032 | RBC (em) |
| O | O | Reticulocyte | L2011 | Reticulocyte |
| O | O | Sodium | L0006 | Sodium (serum) [POCT] |
| O | O | Sodium | L3043 | Sodium (serum) |
| O | O | Sodium | L31241 | Sodium (serum) |
| O | O | Sodium | L8130 | Sodium (serum, em) |
| O | O | Total bilirubin | L3011 | Bilirubin, total |
| O | O | Total bilirubin | L6108 | Bilirubin |
| O | O | Total bilirubin | L8012 | Bilirubin |
| O | O | Total bilirubin | L8136 | Bilirubin, total (em) |
| O | O | Triglyceride | L0040 | Triglyceride (TG) [POCT] |
| O | O | Triglyceride | L3061 | Triglyceride (TG) |
| O |  | Triiodothyronine | L7605 | T3(Total) |
| O |  | Triiodothyronine | L7626 | Free T3 |
|  | O | TSH | L7606 | TSH |
|  | O | TSH | L76106 | TSH |
| O | O | Uric Acid | L3007 | Uric Acid |
| O | O | Uric Acid | L8164 | Uric Acid (em) |
| O |  | Urine blood | L61301 | RBC (u) |
| O |  | Urine blood | L80161 | RBC (u) |
|  | O | Urine calcium | L3104 | Calcium (24hrs urine) |
|  | O | Urine glucose | L3108 | Glucose quan. (24hrs urine) |
| O | O | Urine protein | L3109 | Protein (24hrs urine) |
| O | O | Urine protein | L31090 | Protein (random urin |
| O | O | Urine protein | L311701 | Microalbumin (random |
| O | O | Urine protein | L311702 | Microalbumin/Creatin |
| O | O | Urine protein | L31171 | Microalbumin (24hrs urine) |
| O | O | Urine protein | L31172 | Microalbumin/Creatin |
| O | O | Urine protein | L8009 | Albumin (urine, em) |
| O | O | White blood cell | L2001 | WBC |
| O | O | White blood cell | L61302 | WBC |
| O | O | White blood cell | L62052 | WBC |
| O | O | White blood cell | L8004 | WBC (em) |
| O | O | White blood cell | L80162 | WBC |
| O | O | White blood cell | L80232 | WBC |
| O |  | Urobilinogen | L6110 | Urobilinogen |
| O |  | Urobilinogen | L8014 | Urobilinogen |
|  |  |  |  |  |
