# Supplementary material for: Standard-based comprehensive detection of adverse drug reaction signals from nursing statements and laboratory results in electronic health records
Source: J Am Med Inform Assoc. 2017 Jan 13;24(4):697–708. doi: 10.1093/jamia/ocw168 (PMC7651894; doi:10.1093/jamia/ocw168)
Supplement: Supplementary Data [file ocw168_supp.zip › Supplementary_Table_S3_b_r.docx]

| **Supplementary Table S3** Laboratory tests are mapped to SNUH codes. Laboratory test abnormality lists applied to CLEAR and MetaLAB. **(b)** Forty laboratory tests and 47 laboratory abnormalities applied to CLEAR. | |
| --- | --- |
|  |  |
| **Laboratory test abnormality (*n*=47)** | **Laboratory test name (*n*=40)** |
| activated partial thromboplastin time increased | Activated partial thromboplastin time |
| alanine transaminase increased | Alanine transaminase |
| alkaline phosphatase increased | Alkaline phosphatase |
| ammonemia increased | Ammonemia |
| amylase increased | Amylase |
| aspartate transaminase increased | Aspartate transaminase |
| basophil decreased | Basophil |
| blood urea nitrogen increased | Blood urea nitrogen |
| cholesterol increased | Cholesterol |
| creatine kinase increased | Creatine kinase |
| creatinine increased | Creatinine |
| direct bilirubin increased | Direct bilirubin |
| eosinophil decreased | Eosinophil |
| eosinophil increased |  |
| fibrinogen decreased | Fibrinogen |
| free thyroxine decreased | Free thyroxine |
| free thyroxine increased |  |
| gamma-glutamyl transpeptidase increased | Gamma-glutamyl transpeptidase |
| glucose decreased | Glucose |
| glucose increased |  |
| hematocrit decreased | Hematocrit |
| hemoglobin decreased | Hemoglobin |
| hemoglobin increased |  |
| lactate dehydrogenase increased | Lactate dehydrogenase |
| LDL cholesterol increased | LDL cholesterol |
| lipase increased | Lipase |
| lymphocyte increased | Lymphocyte |
| myoglobin increased | Myoglobin |
| neutrophil decreased | Neutrophil |
| platelet decreased | Platelet |
| platelet increased |  |
| potassium increased | Potassium |
| prolactin increased | Prolactin |
| prothrombin time increased | Prothrombin time |
| red blood cell decreased | Red blood cell |
| reticulocyte decreased | Reticulocyte |
| reticulocyte increased |  |
| sodium decreased | Sodium |
| total bilirubin increased | Total bilirubin |
| triglyceride increased | Triglyceride |
| triiodothyronine decreased | Triiodothyronine |
| triiodothyronine increased |  |
| uric acid increased | Uric Acid |
| urine blood increased | Urine blood |
| urine protein increased | Urine protein |
| urobilinogen increased | Urobilinogen |
| white blood cell decreased | White blood cell |
|  |  |
