# Supplementary material for: Standard-based comprehensive detection of adverse drug reaction signals from nursing statements and laboratory results in electronic health records
Source: J Am Med Inform Assoc. 2017 Jan 13;24(4):697–708. doi: 10.1093/jamia/ocw168 (PMC7651894; doi:10.1093/jamia/ocw168)
Supplement: Supplementary Data [file ocw168_supp.zip › Supplementary_Table_S3_c_r.docx]

| **Supplementary Table S3** Laboratory tests are mapped to SNUH codes. Laboratory test abnormality lists applied to CLEAR and MetaLAB. **(c)** Forty-eight laboratory tests and 117 laboratory abnormalities applied to MetaLAB. | | | | | | |
| --- | --- | --- | --- | --- | --- | --- |
|  |  |  |  |  |  |  |
| **Laboratory test abnormality (MedDRA PTs, *n*=117)** | **High or Low** | **Laboratory test name_1** | **Laboratory test name_2** | **Laboratory test name_3** | **Laboratory test name_4** | **Laboratory test name_5** |
| Acidosis | H/L | pH |  |  |  |  |
| Agranulocytosis | L | Neutrophil | White blood cell |  |  |  |
| Alanine aminotransferase increased | H | Alanine transaminase |  |  |  |  |
| Albuminuria | H | Urine protein |  |  |  |  |
| Alkalosis | H/L | pH |  |  |  |  |
| Alkalosis hypokalaemic | H | pH | Potassium |  |  |  |
| Anaemia | L | Hemoglobin | Hematocrit |  |  |  |
| Anaemia macrocytic | H/L | Hemoglobin | Hematocrit |  |  |  |
| Anaemia megaloblastic | L | Hemoglobin | Hematocrit |  |  |  |
| Aplastic anaemia | L | Hemoglobin | Hematocrit |  |  |  |
| Aspartate aminotransferase increased | H | Aspartate transaminase |  |  |  |  |
| Azotaemia | L | Creatinine Clearance |  |  |  |  |
| Basophilia | H | Basophil |  |  |  |  |
| Blood albumin decreased | L | Albumin |  |  |  |  |
| Blood alkaline phosphatase increased | H | Alkaline phosphatase |  |  |  |  |
| Blood amylase increased | H | Amylase |  |  |  |  |
| Blood bilirubin increased | H | Total bilirubin | Direct bilirubin |  |  |  |
| Blood cholesterol increased | H | Cholesterol |  |  |  |  |
| Blood creatinine abnormal | H/L | Creatinine |  |  |  |  |
| Blood creatinine decreased | L | Creatinine |  |  |  |  |
| Blood creatinine increased | H | Creatinine |  |  |  |  |
| Blood electrolytes abnormal | H/L | Potassium | Sodium | Chloride |  |  |
| Blood electrolytes decreased | L | Potassium | Sodium | Chloride |  |  |
| Blood fibrinogen increased | H | Fibrinogen |  |  |  |  |
| Blood glucose abnormal | H/L | Glucose |  |  |  |  |
| Blood glucose increased | H | Glucose |  |  |  |  |
| Blood insulin increased | L | Insulin |  |  |  |  |
| Blood iron decreased | L | Iron |  |  |  |  |
| Blood potassium decreased | L | Potassium |  |  |  |  |
| Blood potassium increased | H | Potassium |  |  |  |  |
| Blood prolactin increased | H | Prolactin |  |  |  |  |
| Blood triglycerides increased | H | Triglyceride |  |  |  |  |
| Blood triglycerides normal | H/L | Triglyceride |  |  |  |  |
| Blood urea increased | H | Blood urea nitrogen |  |  |  |  |
| Blood uric acid increased | H | Uric Acid |  |  |  |  |
| Creatinine renal clearance decreased | L | Creatinine |  |  |  |  |
| Deficiency anaemia | L | Hemoglobin | Hematocrit |  |  |  |
| Dyslipidaemia | H/L | Cholesterol |  |  |  |  |
| Electrolyte imbalance | H/L | Cholesterol | Potassium | Sodium | Chloride |  |
| Eosinophil count increased | H | Eosinophil |  |  |  |  |
| Eosinophilia | H | Eosinophil |  |  |  |  |
| Febrile neutropenia | L | Neutrophil |  |  |  |  |
| Glomerular filtration rate decreased | L | Creatinine Clearance |  |  |  |  |
| Glycosuria | H | Urine glucose |  |  |  |  |
| Haematocrit decreased | L | Hematocrit |  |  |  |  |
| Haematocrit increased | H | Hematocrit |  |  |  |  |
| Haemoglobin | H/L | Hemoglobin | Hematocrit |  |  |  |
| Haemoglobin abnormal | H/L | Hemoglobin | Hematocrit |  |  |  |
| Haemoglobin decreased | L | Hemoglobin | Hematocrit |  |  |  |
| Haemoglobin increased | H | Hemoglobin | Hematocrit |  |  |  |
| Haemolytic anaemia | L | Hemoglobin | Hematocrit |  |  |  |
| Hepatic enzyme increased | H | Aspartate transaminase | Alanine transaminase | Gamma-glutamyl transpeptidase | Total bilirubin | Direct bilirubin |
| Hepatic function abnormal | H/L | Alanine transaminase | Aspartate transaminase | Total bilirubin | Direct bilirubin |  |
| High density lipoprotein decreased | L | LDL cholesterol |  |  |  |  |
| Hormone level abnormal | H/L | TSH |  |  |  |  |
| Hyperammonaemia | H | Ammonia |  |  |  |  |
| Hyperbilirubinaemia | H | Total bilirubin | Direct bilirubin |  |  |  |
| Hypercalcaemia | H | Calcium | Calcium, ionized |  |  |  |
| Hypercalciuria | H | Urine calcium |  |  |  |  |
| Hyperchloraemia | H | Chloride |  |  |  |  |
| Hypercholesterolaemia | H | Cholesterol |  |  |  |  |
| Hyperglycaemia | H | Glucose |  |  |  |  |
| Hyperinsulinism | H | Insulin |  |  |  |  |
| Hyperkalaemia | H | Potassium |  |  |  |  |
| Hyperlipidaemia | H | Cholesterol |  |  |  |  |
| Hypermagnesaemia | H | Magnesium |  |  |  |  |
| Hypernatraemia | H | Sodium |  |  |  |  |
| Hyperphosphataemia | H | Phosphorus |  |  |  |  |
| Hyperprolactinaemia | H | Prolactin |  |  |  |  |
| Hypertriglyceridaemia | H | Triglyceride |  |  |  |  |
| Hyperuricaemia | H | Uric Acid |  |  |  |  |
| Hypoalbuminaemia | L | Albumin |  |  |  |  |
| Hypocalcaemia | L | Calcium | Calcium, ionized |  |  |  |
| Hypochloraemia | L | Chloride |  |  |  |  |
| Hypocholesterolaemia | L | Cholesterol |  |  |  |  |
| Hypochromic anaemia | L | Hemoglobin | Hematocrit |  |  |  |
| Hypoglycaemia | L | Glucose |  |  |  |  |
| Hypokalaemia | L | Potassium |  |  |  |  |
| Hypomagnesaemia | L | Magnesium |  |  |  |  |
| Hyponatraemia | L | Sodium |  |  |  |  |
| Hypophosphataemia | L | Phosphorus |  |  |  |  |
| Hypoproteinaemia | L | Albumin |  |  |  |  |
| Hypouricaemia | L | Uric Acid |  |  |  |  |
| International normalised ratio increased | H | Prothrombin time |  |  |  |  |
| Iron deficiency | L | Iron |  |  |  |  |
| Iron deficiency anaemia | L | Iron | Hemoglobin | Hematocrit | Reticulocyte |  |
| Leukocytosis | H | Neutrophil |  |  |  |  |
| Leukopenia | L | Neutrophil |  |  |  |  |
| Lipase increased | H | Lipase |  |  |  |  |
| Liver function test abnormal | H/L | Aspartate transaminase | Alanine transaminase | Gamma-glutamyl transpeptidase | Total bilirubin | Direct bilirubin |
| Low density lipoprotein increased | H | LDL cholesterol |  |  |  |  |
| Lymphocyte count abnormal | H/L | Lymphocyte |  |  |  |  |
| Lymphocytosis | H | Lymphocyte |  |  |  |  |
| Lymphopenia | L | Lymphocyte |  |  |  |  |
| Metabolic acidosis | L | pH |  |  |  |  |
| Microcytic anaemia | H/L | Hemoglobin | Hematocrit |  |  |  |
| Monocytosis | H | Monocyte |  |  |  |  |
| Myoglobinuria | H | Myoglobin |  |  |  |  |
| Neutropenia | L | Neutrophil |  |  |  |  |
| Neutrophil count increased | H | Neutrophil |  |  |  |  |
| Normochromic normocytic anaemia | L | Hemoglobin | Hematocrit |  |  |  |
| Pancytopenia | L | Basophil | Eosinophil | Neutrophil | Red blood cell |  |
| Platelet count decreased | L | Platelet |  |  |  |  |
| Protein urine present | H | Urine protein |  |  |  |  |
| Proteinuria | H | Urine protein |  |  |  |  |
| Red blood cell abnormality | H/L | Red blood cell |  |  |  |  |
| Red blood cell sedimentation rate increased | H | ESR |  |  |  |  |
| Reticulocytosis | H | Reticulocyte |  |  |  |  |
| Thrombocytopenia | L | Platelet |  |  |  |  |
| Thrombocytosis | H | Platelet |  |  |  |  |
| Transaminases increased | H | Alanine transaminase | Aspartate transaminase |  |  |  |
| Urine abnormality | H | Urine protein |  |  |  |  |
| Urine analysis abnormal | H | Urine protein |  |  |  |  |
| White blood cell count abnormal | H/L | White blood cell |  |  |  |  |
| White blood cell count decreased | L | White blood cell |  |  |  |  |
| White blood cell count increased | H | White blood cell |  |  |  |  |
| White blood cell disorder | H/L | White blood cell |  |  |  |  |
|  |  |  |  |  |  |  |
