# Supplementary material for: Standard-based comprehensive detection of adverse drug reaction signals from nursing statements and laboratory results in electronic health records
Source: J Am Med Inform Assoc. 2017 Jan 13;24(4):697–708. doi: 10.1093/jamia/ocw168 (PMC7651894; doi:10.1093/jamia/ocw168)
Supplement: Supplementary Data [file ocw168_supp.zip › Supplementary_Table_S4.docx]

**Supplementary Table S4(a)** Bisacodyl related adverse drug reaction of cardiac disorder reports submitted to FDA FAERS database

|  |  |  | |  | | With known cardiac side effect | | | | Without known cardiac side effect | | | |
| --- | --- | --- | --- | --- | --- | --- | --- | --- | --- | --- | --- | --- | --- |
|  | Drug | **Bisacodyl** | | **(1) All drugs in FAERS** | | **(2) Docusate sodium** | | **(3) Polyethylene Glycol 3350** | | **(4) Lactulose** | | **(5) Senna** | |
|  |  | A06AB02 | |  | | A06AA02 | | A06AD15 | | A06AB04 | | A06AB06 | |
|  | Adverse drug reaction | No. of Reports | % | No. of Reports | % | No. of Reports | % | No. of Reports | % | No. of Reports | % | No. of Reports | % |
| 1 | Cardiac failure congestive | 55 | 0.38 | 203,727 | *0.32* | 497 | **0.44** | 251 | *0.29* | 154 | *0.25* | 140 | *0.29* |
| 2 | Atrial fibrillation | 49 | 0.33 | 167,348 | *0.27* | 439 | **0.39** | 220 | *0.26* | 151 | *0.24* | 161 | **0.34** |
| 3 | Cardio-respiratory arrest | 31 | 0.21 | 75,003 | *0.12* | 167 | *0.15* | 75 | *0.09* | 77 | *0.12* | 50 | *0.1* |
| 4 | Cardiac arrest | 25 | 0.17 | 132,803 | **0.21** | 235 | **0.21** | 105 | *0.12* | 154 | **0.25** | 89 | **0.19** |
| 5 | Cardiac failure | 22 | 0.15 | 99,695 | **0.16** | 103 | *0.09* | 38 | *0.04* | 99 | **0.16** | 61 | *0.13* |
| 6 | Cardiomegaly | 20 | 0.14 | 53,116 | *0.08* | 160 | 0.14 | 82 | *0.10* | 69 | *0.11* | 37 | *0.08* |
| 7 | Arrhythmia | 17 | 0.12 | 70,255 | *0.11* | 120 | *0.11* | 64 | *0.08* | 49 | *0.08* | 34 | *0.07* |
| 8 | Atrial flutter | 13 | 0.09 | 19,298 | *0.03* | 60 | *0.05* | 31 | *0.04* | 18 | *0.03* | 21 | *0.04* |
| 9 | Cardiac disorder | 13 | 0.09 | 111,165 | **0.18** | 149 | **0.13** | 78 | 0.09 | 36 | *0.06* | 43 | 0.09 |
| 10 | Ventricular tachycardia | 11 | 0.08 | 37,575 | *0.06* | 94 | 0.08 | 44 | *0.05* | 43 | *0.07* | 37 | 0.08 |
| 11 | Ventricular fibrillation | 9 | 0.06 | 24,582 | *0.04* | 42 | *0.04* | 17 | *0.02* | 26 | *0.04* | 14 | *0.03* |
| 12 | Cardiomyopathy | 9 | 0.06 | 35,731 | 0.06 | 82 | **0.07** | 56 | **0.07** | 25 | *0.04* | 24 | *0.05* |
| 13 | Cardiogenic shock | 9 | 0.06 | 20,200 | *0.03* | 57 | *0.05* | 7 | *0.01* | 17 | *0.03* | 9 | *0.02* |
| 14 | Ventricular extrasystoles | 8 | 0.05 | 29,631 | 0.05 | 73 | **0.06** | 35 | *0.04* | 31 | 0.05 | 40 | **0.08** |
| 15 | Dyspnoea exertional | 8 | 0.05 | 51,772 | **0.08** | 122 | **0.11** | 72 | **0.08** | 65 | **0.11** | 46 | **0.1** |
| 16 | Cardiac murmur | 7 | 0.05 | 29,774 | 0.05 | 78 | **0.07** | 42 | 0.05 | 41 | **0.07** | 20 | *0.04* |
| 17 | Cardiovascular disorder | 6 | 0.04 | 42,131 | **0.07** | 41 | 0.04 | 11 | *0.01* | 30 | **0.05** | 13 | *0.03* |
| 18 | Atrial tachycardia | 5 | 0.03 | 5,022 | *0.01* | 7 | *0.01* | 7 | *0.01* | 2 | *0.00* | 5 | *0.01* |
| 19 | Ventricular hypertrophy | 4 | 0.03 | 15,547 | *0.02* | 38 | 0.03 | 10 | *0.01* | 21 | 0.03 | 15 | 0.03 |
| 20 | Ventricular hypokinesia | 3 | 0.02 | 11,120 | 0.02 | 28 | 0.02 | 0 | *0.00* | 15 | 0.02 | 9 | 0.02 |
| 21 | Cardiac asthma | 3 | 0.02 | 543 | *0* | 0 | *0.00* | 2 | *0.00* | 2 | *0* | 2 | *0.00* |
| 22 | Cardiac failure acute | 3 | 0.02 | 7,427 | *0.01* | 4 | *0.00* | 2 | *0.00* | 7 | *0.01* | 7 | *0.01* |
| 23 | Cardioactive drug level increased | 3 | 0.02 | 8,518 | *0.01* | 20 | 0.02 | 15 | 0.02 | 8 | *0.01* | 10 | 0.02 |
| 24 | Cardiac valve disease | 3 | 0.02 | 13,442 | 0.02 | 29 | **0.03** | 15 | 0.02 | 12 | 0.02 | 10 | 0.02 |
| 25 | Ventricular dysfunction | 2 | 0.01 | 9,307 | 0.01 | 29 | **0.03** | 6 | 0.01 | 11 | **0.02** | 4 | 0.01 |
| 26 | Cardiac tamponade | 2 | 0.01 | 6,555 | 0.01 | 13 | 0.01 | 1 | *0.00* | 1 | *0.00* | 7 | 0.01 |
| 27 | Dyspnoea paroxysmal nocturnal | 2 | 0.01 | 4,491 | 0.01 | 18 | **0.02** | 9 | 0.01 | 3 | *0.00* | 9 | **0.02** |
| 28 | Atrial hypertrophy | 1 | 0.01 | 487 | *0.00* | 1 | *0.00* | 0 | *0.00* | 2 | *0.00* | 0 | *0.00* |
| 29 | Cardioactive drug level above therapeutic | 1 | 0.01 | 2,092 | *0.00* | 12 | 0.01 | 3 | *0.00* | 2 | *0.00* | 2 | *0.00* |
| 30 | Cardiorenal syndrome | 1 | 0.01 | 797 | *0.00* | 5 | *0.00* | 3 | *0.00* | 2 | *0.00* | 1 | *0.00* |
| 31 | Cardiopulmonary failure | 1 | 0.01 | 7,063 | 0.01 | 11 | 0.01 | 4 | *0.00* | 17 | **0.03** | 2 | *0.00* |
| 32 | Cardiac pacemaker replacement | 1 | 0.01 | 584 | *0.00* | 2 | *0.00* | 1 | *0.00* | 0 | *0.00* | 1 | *0.00* |
| 33 | Cardiolipin antibody positive | 1 | 0.01 | 776 | *0.00* | 2 | *0.00* | 1 | *0.00* | 3 | *0.00* | 0 | *0.00* |
| 34 | Cardiac pacemaker insertion | 1 | 0.01 | 9,845 | **0.02** | 17 | **0.02** | 12 | 0.01 | 0 | *0.00* | 7 | 0.01 |
| 35 | Cardiac flutter | 1 | 0.01 | 8,257 | 0.01 | 17 | **0.02** | 9 | 0.01 | 0 | 0.01 | 5 | 0.01 |
| 36 | Cardiac output decreased | 1 | 0.01 | 2,189 | *0.00* | 9 | 0.01 | 2 | *0.00* | 4 | 0.01 | 4 | 0.01 |
| 37 | Cardiac failure chronic | 1 | 0.01 | 3,959 | 0.01 | 4 | 0.00 | 2 | *0.00* | 3 | *0.00* | 6 | 0.01 |
| 38 | Cardiac myxoma | 1 | 0.01 | 315 | *0.00* | 1 | 0.00 | 0 | *0.00* | 0 | *0.00* | 0 | *0.00* |
| 39 | Conduction disorder | 1 | 0.01 | 4,668 | 0.01 | 8 | 0.01 | 8 | 0.01 | 5 | 0.01 | 2 | *0.00* |
|  | Total | 354 | **2.44** | 1,326,810 | ***2.10*** | 2,794 | **2.48** | 1,340 | ***1.55*** | 1,205 | ***1.93*** | 947 | ***1.95*** |

**Supplementary Table S4 (b)** The background rate of events in the FAERS data for metabolism and nutrition disorders related to prazosin

|  |  | **Prazosin** | | **All drugs in FAERS** | | |
| --- | --- | --- | --- | --- | --- | --- |
|  | **Adverse drug reaction** | **No. of Reports** | **%** | **No. of Reports** | **%** |  |
| 1 | Hyperkalaemia | 33 | 0.44 | 66,242 | 0.1 |  |
| 2 | weight decreased | 29 | 0.38 | 318,798 | **0.5** |  |
| 3 | Dehydration | 26 | 0.34 | 233,853 | **0.37** |  |
| 4 | decreased appetite | 19 | 0.25 | 275,825 | **0.44** |  |
| 5 | Hypernatraemia | 2 | 0.03 | 8,386 | 0.01 |  |
| 6 | hypophosphataemia | 1 | 0.01 | 10,183 | **0.02** |  |
|  |  | **110** | **1.45** | **913,287** | **1.44** |  |

**Supplementary Table S4 (c)** The background rate of events in the FAERS data for psychiatric disorders related to phenylephrine

|  |  | **Phenylephrine** | | **All drugs in FAERS** | |
| --- | --- | --- | --- | --- | --- |
|  | **Adverse drug reaction** | **No. of Reports** | **%** | **No. of Reports** | **%** |
| 1 | emotional distress | 189 | 2.03 | 113,556 | 0.18 |
| 2 | depression | 93 | 1.00 | 330,104 | 0.52 |
| 3 | fear of death | 23 | 0.25 | 4,725 | 0.01 |
| 4 | confusional state | 21 | 0.23 | 221,684 | **0.35** |
| 5 | mental status changes | 21 | 0.23 | 69,133 | 0.11 |
| 6 | psychiatric symptom | 13 | 0.14 | 8,773 | 0.01 |
| 7 | somnolence | 10 | 0.11 | 208,123 | **0.33** |
| 8 | mental disorder | 10 | 0.11 | 51,973 | 0.08 |
| 9 | delirium | 8 | 0.09 | 43,757 | 0.07 |
| 10 | disorientation | 4 | 0.04 | 63,771 | 0.1 |
| 11 | mental impairment | 3 | 0.03 | 25,586 | **0.04** |
| 12 | confusion postoperative | 1 | 0.01 | 500 | 0 |
| 13 | mental disorder due to a general medical condition | 1 | 0.01 | 1,152 | 0 |
| 14 | consciousness fluctuating | 1 | 0.01 | 1,317 | 0 |
|  |  | **398** | **4.29** | **1,144,154** | **1.8** |

**Supplementary Table S4 (d)** The background rate of events in the FAERS data for renal and urinary disorders related to sucralfate

|  |  | **Sucralfate** | | **All drugs in FAERS** | |
| --- | --- | --- | --- | --- | --- |
|  | **Adverse drug reaction** | **No. of Reports** | **%** | **No. of Reports** | **%** |
| 1 | urinary tract infection | 130 | 0.37 | 199,424 | 0.32 |
| 2 | renal failure | 129 | 0.37 | 226,371 | 0.36 |
| 3 | renal failure acute | 119 | 0.34 | 235,625 | **0.37** |
| 4 | renal failure chronic | 34 | 0.10 | 56,292 | 0.09 |
| 5 | renal disorder | 29 | 0.08 | 49,455 | 0.08 |
| 6 | renal impairment | 27 | 0.08 | 103,372 | **0.16** |
| 7 | urinary retention | 23 | 0.07 | 44,025 | 0.07 |
| 8 | renal cyst | 21 | 0.06 | 23,836 | 0.04 |
| 9 | urinary incontinence | 20 | 0.06 | 46,987 | **0.07** |
| 10 | renal tubular necrosis | 16 | 0.05 | 19,850 | 0.03 |
| 11 | urine output decreased | 12 | 0.03 | 18,662 | 0.03 |
| 12 | renal injury | 11 | 0.03 | 31,729 | **0.05** |
| 13 | chromaturia | 9 | 0.03 | 25,794 | **0.04** |
| 14 | azotaemia | 8 | 0.02 | 11,535 | 0.02 |
| 15 | tubulointerstitial nephritis | 7 | 0.02 | 17,822 | **0.03** |
| 16 | oliguria | 7 | 0.02 | 12,248 | 0.02 |
| 17 | renal cell carcinoma | 7 | 0.02 | 9,677 | 0.02 |
| 18 | urinary hesitation | 6 | 0.02 | 4,709 | 0.01 |
| 19 | urinary tract disorder | 6 | 0.02 | 4,831 | 0.01 |
| 20 | proteinuria | 5 | 0.01 | 23,110 | **0.04** |
| 21 | bladder disorder | 4 | 0.01 | 12,730 | **0.02** |
| 22 | renal function test abnormal | 4 | 0.01 | 3,969 | 0.01 |
| 23 | renal mass | 4 | 0.01 | 3,569 | 0.01 |
| 24 | renal artery stenosis | 4 | 0.01 | 4,846 | 0.01 |
| 25 | renal cancer | 4 | 0.01 | 7,170 | 0.01 |
| 26 | urine analysis abnormal | 3 | 0.01 | 5,020 | 0.01 |
| 27 | urinary tract obstruction | 3 | 0.01 | 3,246 | 0.01 |
| 28 | bladder prolapse | 3 | 0.01 | 2,749 | 0 |
| 29 | renal haemorrhage | 3 | 0.01 | 2,670 | 0 |
| 30 | renal transplant | 3 | 0.01 | 4,682 | 0.01 |
| 31 | urine output increased | 2 | 0.01 | 3,459 | 0.01 |
| 32 | nephritis | 2 | 0.01 | 2,884 | 0 |
| 33 | urinary tract infection pseudomonal | 2 | 0.01 | 1,761 | 0 |
| 34 | bladder spasm | 2 | 0.01 | 3,030 | 0 |
| 35 | bladder cancer | 2 | 0.01 | 13,674 | **0.02** |
| 36 | ureteric obstruction | 2 | 0.01 | 3,102 | 0 |
| 37 | renal cancer metastatic | 2 | 0.01 | 1,179 | 0 |
| 38 | renal artery arteriosclerosis | 2 | 0.01 | 495 | 0 |
| 39 | renal tubular acidosis | 2 | 0.01 | 2,500 | 0 |
| 40 | urethral stenosis | 1 | 0.00 | 1,908 | 0 |
| 41 | urine colour abnormal | 1 | 0.00 | 2,281 | 0 |
| 42 | urine chloride decreased | 1 | 0.00 | 82 | 0 |
| 43 | urine bilirubin increased | 1 | 0.00 | 391 | 0 |
| 44 | urine calcium decreased | 1 | 0.00 | 111 | 0 |
| 45 | urinary bladder haemorrhage | 1 | 0.00 | 2,679 | 0 |
| 46 | urinary bladder rupture | 1 | 0.00 | 225 | 0 |
| 47 | urinary tract infection fungal | 1 | 0.00 | 1,455 | 0 |
| 48 | glomerulonephritis | 1 | 0.00 | 3,609 | **0.01** |
| 49 | glomerulonephritis proliferative | 1 | 0.00 | 619 | 0 |
| 50 | bladder pain | 1 | 0.00 | 3,020 | 0 |
| 51 | bladder neoplasm | 1 | 0.00 | 2,281 | 0 |
| 52 | bladder dilatation | 1 | 0.00 | 2,161 | 0 |
| 53 | bladder diverticulum | 1 | 0.00 | 494 | 0 |
| 54 | bladder obstruction | 1 | 0.00 | 1,294 | 0 |
| 55 | bladder irritation | 1 | 0.00 | 601 | 0 |
| 56 | urge incontinence | 1 | 0.00 | 1,748 | 0 |
| 57 | renal tubular disorder | 1 | 0.00 | 5,953 | **0.01** |
| 58 | renal stone removal | 1 | 0.00 | 531 | 0 |
| 59 | renal necrosis | 1 | 0.00 | 610 | 0 |
| 60 | renal pain | 1 | 0.00 | 9,265 | 0.01 |
| 61 | renal haematoma | 1 | 0.00 | 766 | 0 |
| 62 | renal cortical necrosis | 1 | 0.00 | 318 | 0 |
| 63 | renal atrophy | 1 | 0.00 | 4,261 | **0.01** |
| 64 | renal arteriosclerosis | 1 | 0.00 | 766 | 0 |
| 65 | renal ischaemia | 1 | 0.00 | 856 | 0 |
|  |  | **705** | **1.99** | **1,296,374** | **2.02** |
