# Supplementary material for: Standard-based comprehensive detection of adverse drug reaction signals from nursing statements and laboratory results in electronic health records
Source: J Am Med Inform Assoc. 2017 Jan 13;24(4):697–708. doi: 10.1093/jamia/ocw168 (PMC7651894; doi:10.1093/jamia/ocw168)
Supplement: Supplementary Data [file ocw168_supp.zip › Supplementary_Table_S5.docx]

**Supplementary Table S5. (a)** Variables for obtaining AUC values for each drug.

| **Input variable** | | | | | | | **Outcome**  **(SIDER2)** | **AUC** |
| --- | --- | --- | --- | --- | --- | --- | --- | --- |
| **Drug** | **ADR** | | | **Odds ratio** | **P** | **Adjusted P** |  |  |
|  | **MedDRA PT** | **Lab test 1** | **Lab test 2** |  |  |  |  |  |
| Atropine | Hypochoromic anaemia | Hematocrit | Hemoglobin | 2.078 | 0.366 | 0.491 | 1 | 0.69 |
| Atropine | Leukocytosis | Neutrophil |  | 1.310 | 0.067 | 0.146 | 1 |  |
| … | … | … | … | … |  | … | … |  |
|  |  |  |  |  |  |  |  |  |
|  |  |  |  |  |  |  |  |  |

**Supplementary Table S5. (b)** Calculating AUC values per each SOC subgroup to obtain 'SOC-integrated' AUC values to reduce the number of spurious negative associations in the reference set from SIDER 2.

| **Input variable** | | | | | | | **Outcome**  **(SIDER)** | **AUC** |
| --- | --- | --- | --- | --- | --- | --- | --- | --- |
| **SOC** | **Drug** | **ADR** | | | **Odds ratio** | **Adjusted *p*-value** |  |  |
|  |  | **MedDRA PT** | **Lab test 1** | **Lab test 2** |  |  |  |  |
| Blood and lymphatic system disorders | Atropine | Hypochoromic anaemia | Hematocrit | Hemoglobin | 2.078 | 0.491 | 1 | 0.69 |
| Blood and lymphatic system disorders | Atropine | Leukocytosis | Neutrophil |  | 1.310 | 0.146 | 1 |  |
|  | … | … | … | … | … | … | … |  |
| Blood and lymphatic system disorders | famotidine | Hypochoromic anaemia | Hematocrit | Hemoglobin | 3.361 | 0.435 | 1 | 0.84 |
| Blood and lymphatic system disorders | famotidine | Leukocytosis | Neutrophil |  | 1.988 | 0.011 | 0 |  |
|  | … | … | … | … | … | … | … |  |
